# CATH-ddG: towards robust mutation effect prediction on protein–protein interactions out of CATH homologous superfamily

**DOI:** 10.1093/bioinformatics/btaf228

**Published:** 2025-07-15

**Authors:** Guanglei Yu, Xuehua Bi, Teng Ma, Yaohang Li, Jianxin Wang

**Affiliations:** School of Computer Science and Engineering, Central South University, Changsha 410083, China; Hunan Provincial Key Lab on Bioinformatics, Central South University, Changsha 410083, China; College of Medical Engineering and Technology, Xinjiang Medical University, Urumqi 830017, China; College of Medical Engineering and Technology, Xinjiang Medical University, Urumqi 830017, China; School of Computer Science and Engineering, Central South University, Changsha 410083, China; Hunan Provincial Key Lab on Bioinformatics, Central South University, Changsha 410083, China; Department of Computer Science, Old Dominion University, Norfolk, VA 23529, United States; School of Computer Science and Engineering, Central South University, Changsha 410083, China; Hunan Provincial Key Lab on Bioinformatics, Central South University, Changsha 410083, China

## Abstract

**Motivation:**

Protein–protein interactions (PPIs) are fundamental aspects in understanding biological processes. Accurately predicting the effects of mutations on PPIs remains a critical requirement for drug design and disease mechanistic studies. Recently, deep learning models using protein 3D structures have become predominant for predicting mutation effects. However, significant challenges remain in practical applications, in part due to the considerable disparity in generalization capabilities between easy and hard mutations. Specifically, a hard mutation is defined as one with its maximum TM-score <0.6 when compared to the training set. Additionally, compared to physics-based approaches, deep learning models may overestimate performance due to potential data leakage.

**Results:**

We propose new training/test splits that mitigate data leakage according to the CATH homologous superfamily. Under the constraints of physical energy, protein 3D structures, and CATH domain objectives, we employ a hybrid noise strategy as data augmentation and present a geometric encoder scenario, named CATH-ddG, to represent the mutational microenvironment differences between wild-type and mutated protein complexes. Additionally, we fine-tune ESM2 representations by incorporating a lightweight nonlinear module to achieve the transferability of sequence co-evolutionary information. Finally, our study demonstrates that CATH-ddG framework provides enhanced generalization by outperforming other baselines on non-superfamily leakage splits, which plays a crucial role in exploring robust mutation effect regression prediction. Independent case studies demonstrate successful enhancement of binding affinity on 419 antibody variants to human epidermal growth factor receptor 2 (HER2) and 285 variants in the receptor-binding domain (RBD) of SARS-CoV-2 to angiotensin-converting enzyme 2 (ACE2) receptor.

**Availability and implementation:**

CATH-ddG is available at https://github.com/ak422/CATH-ddG.

## 1 Introduction

Efforts have been dedicated to gleaning biological and therapeutic insights directly from disease-associated genomic sequencing. Crucially, protein–protein interactions (PPIs) and their resultant molecular machines are involved in virtually all cellular functions. Disease-related mutations often perturb protein functions/interactions, as well as the majority of therapeutics that are designed to target proteins. Thus, the deep mechanistic understanding of PPI maps and their associated mutations is crucial for comprehending healthy biology and ultimately treating disease (Greenblatt *et al.* 2024).

Traditional and experimental evaluations of the mutation effects on PPIs face inherent limitations related to experimental complexity and throughput. For instance, flex ddG is the state-of-the-art (SOTA) Rosetta protocol (Barlow *et al.* 2018), which estimates the change in binding free energy (ΔΔG) between wild-type and mutant structures by considering conformational plasticity through sampling and using the “backrub” protocol. Nevertheless, in terms of speed, they are inapplicable for mutation screening, which is five orders of magnitude slower than machine learning methods (Bushuiev *et al.* 2024). In contrast, by utilizing empirical effective energy functions (EEEFs), the FoldX (Delgado *et al.* 2019) enables rapid and quantitative evaluation of mutation effect on the stability, folding, and dynamics of proteins and protein complexes. The predictive performance of FoldX is assessed on large-scale mutations, and applied to evaluate the reliability of protein engineering applications. Therefore, we hypothesize that the prior knowledge of energy effects derived from FoldX can be integrated into deep neural networks to enable fast and accurate prediction of ΔΔG.

In recent years, self-supervised protein language models (PLMs) have had a tremendous impact on the protein community. PLMs have emerged as potent tools for designing protein structures and predicting functions. While using PLMs can provide guidance on modeling fitness landscapes in a zero-shot or fine-tune manner, particularly for predicting the mutation effect of single proteins (Meier *et al.* 2021, Diaz *et al.* 2024), these approaches struggle to generalize across diverse protein families to rank mutation effects (Hie *et al.* 2024, Jiang *et al.* 2025). In addition, directly extending PLMs to predict the mutation effects on PPIs is challenging, mainly due to the lack of co-evolutionary information caused by proteins that may belong to different species in protein complexes (Luo *et al.* 2023), such as host–pathogen interactions.

Meanwhile, structure-based models can capture interactions within proteins and with other biological molecules (such as ligands, nucleotides, or other proteins), which makes them more effective in predicting the mutation effects than sequence-only methods. The end-to-end computational approaches, such as DDGPred (Shan *et al.* 2022), enable the direct prediction of ΔΔG by utilizing a geometric encoder to extract features of inter-residue interactions from protein three-dimensional (3D) structures, and simultaneously incorporating physical energy terms from Rosetta (Alford *et al.* 2017) as shared features across residues.

In comparison, most recent structure-based methods (Liu *et al.* 2023, Luo *et al.* 2023, Bushuiev *et al.* 2024, Cai *et al.* 2024, Wu *et al.* 2024) leverage a pre-training strategy on protein 3D structures to learn protein structure representations, and then transfer the learned knowledge to downstream ΔΔG prediction. For example, RDE-Network (Luo *et al.* 2023) employs a normalizing flow-based generative model to estimate the probability distribution of protein side-chain conformations. Similarly, DiffAffinity (Liu *et al.* 2023) presents a pre-trained Riemannian diffusion probabilistic model to learn the generative process of side-chain conformations. Aiming at modeling the joint distribution of mutations, residue types, angular statistics, and local conformations in the microenvironment, Prompt-DDG (Wu *et al.* 2024) constructs a pre-trained prompt codebook to quantify the microenvironmental differences between wild-type and mutant protein complexes.

Despite the above-mentioned fruitful progress, the efficacy of existing structure-based pre-training predictors is predominantly limited by the high variance and data leakage. Firstly, compared to physics-based approaches such as FoldX and flex ddG (Barlow *et al.* 2018), deep learning models perform superior performance on easy mutations, but their generalization capabilities perform poorly when dealing with hard mutations (Cai *et al.* 2024). Secondly, current training/test splitting strategies suffer from at least three levels of data leakage issues, including mutation-level, protein-level, and complex-level (see Section 2 for details), which are recognized as critical issues hindering meaningful progress in the protein community. Moreover, pre-training tasks achieve superior performance, but generally bring large computational cost.

To address these issues, on the one hand, we curate a training/test splitting strategy based on the CATH (Sillitoe *et al.* 2021) homologous superfamily to mitigate data leakage. The CATH is a protein domain classification database, where domains are curated automatically and manually by experts to construct hierarchical classifications of evolutionary and structural relationships. Currently, CATH v4.4 includes over 600 000 experimentally determined domain structures from PDB, which are categorized into 6573 superfamilies. Consequently, according to the assumption that specific protein function stems from domain-specific interactions, we employ a multi-label self-supervised learning task to effectively capture the domain annotation constraints in protein complexes.

On the other hand, we hypothesize that combining PLMs with protein 3D structures will allow for the simultaneous identification of multiple high binding affinity regions, as well as the generalization across diverse protein homologous families with rugged binding affinity landscape while ensuring robustness to local optima.

Specifically, we present a lightweight nonlinear module, akin to AdaptFormer (Chen *et al.* 2022), to fine-tune ESM2 (Lin *et al.* 2023), and achieve transferability of ESM2 without updating its original pre-trained parameters.

Summarized briefly, our contributions are listed as follows:

1. introducing new training/test splits to mitigate data leakage at the level of CATH homologous superfamily;
2. proposing a novel structure-based geometric deep learning model (CATH-ddG) for ΔΔG prediction, which can capture not only 3D structure information through spatial and sequential geometric encoders, but also domain information of protein complexes, as well as the robustness guided by energy terms of force field method FoldX;
3. fine-tuning ESM2 with a lightweight nonlinear module to achieve transferability of sequence co-evolutionary information;
4. providing systematic and novelly designed improvements to the way of mutant structures generation, data augmentation, node and edge features, and the model architecture.

## 2 Materials and methods

### 2.1 Materials

In this work, for ΔΔG prediction of protein complexes using CATH-ddG, we employ the largest labeled dataset SKEMPI v2.0 (Jankauskaitė *et al.* 2019), which contains 352 PPIs (i.e. #Pdb column in SKEMPI v2.0) and 7085 mutations. After filtering out unavailable PPIs, which include 287 mutations with missing experimental ΔΔG values and 92 mutations with problematic structure in PDB ID: 1KBH (Luo *et al.* 2023), the remaining 343 PPIs and 6706 mutations are used as the benchmark dataset (see Supplementary Section S1.1 for details).

The existing data splitting on PPIs has two kinds of data leakages, which severely impede the generalizability when it comes to unseen binding partners (Bushuiev *et al.* 2024). First, PPIs in the test set have the same mutations as those in training set. For instance, 2WPT_A_B and 1EMV_A_B represent the same interaction between an immune protein of *Escherichia coli* and colicin toxin, with 21 shared mutations to both PPIs, are split into in different folds, which results in data leakage issue at the mutation-level. Second, data splitting on PPIs violates the held-out proteins proposed by SKEMPI v2.0, resulting in the same homologous PPIs Hold_out_proteins between training and test sets. For example, 1CSE_E_I and 1ACB_E_I have six identical mutations of protein Eglin c, which demonstrates the data leakage issue at the protein-level.

Furthermore, we find that different binding partners imply different PPIs, meaning that the same protein complexes with different binding partners are split into training and test sets separately. For instance, the mutations in 2C5D_A_C and 2C5D_AB_CD, 3SE3_B_A and 3SE3_B_C, 3SE4_B_A and 3SE4_B_C involve different PPIs with the same complex, which leads to the data leakage issue at the complex-level.

**Figure 1.**
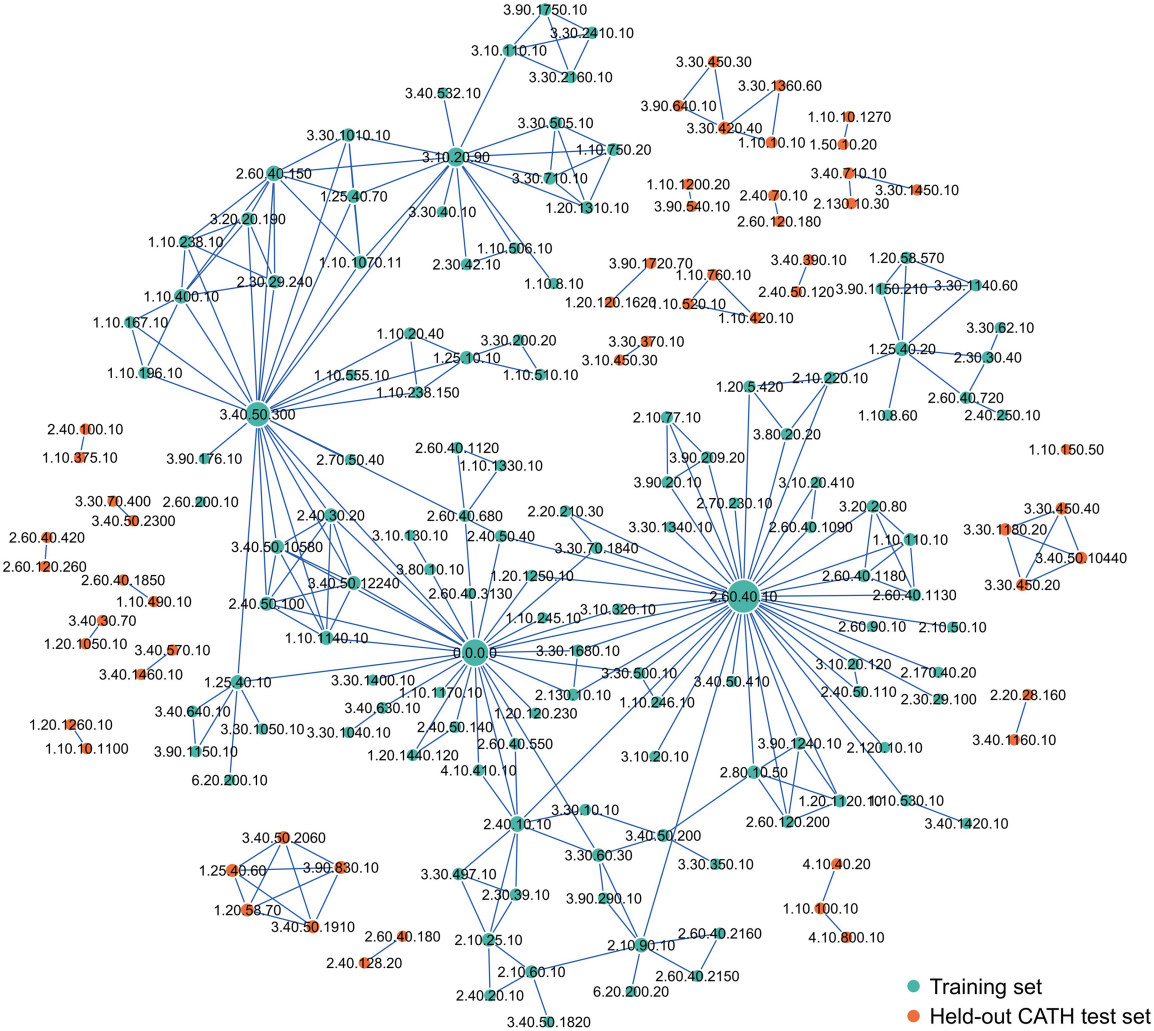
Overview of data splitting for SKEMPI v2.0 according to CATH homologous superfamily. The graph is composed of 179 nodes across 24 connected components, where each node represents a CATH superfamily. Two nodes are connected when a protein complex containing domains from both superfamilies can be found. Node 0.0.0.0 indicates that the protein chain is unannotated, and the most connected superfamily 2.60.40.10 is immunoglobulin-like in SKEMPI v2.0 dataset. The training set consists of 125 CATH superfamily nodes, and the held-out CATH test set consists of 54 CATH superfamily nodes.

PPIFORMER (Bushuiev *et al.* 2024) manually selected five unique Hold_out_proteins, including 14 PPIs and 219 mutations, as a held-out test set. However, it has the limitation that the held-out test set comprises only 4.08% of PPIs and 3.27% of mutations compared to SKEMPI v2.0, which cannot completely and effectively assess the generalization performance of baselines. Thus, based on the CATH homologous superfamilies of protein complexes, as well as putative domain superfamilies of unannotated chains using Foldseek (Van Kempen *et al.* 2024) for structural similarity matches, we divide SKEMPI v2.0 into training set and held-out CATH test set, as shown in Fig. 1. Naturally, this CATH superfamily split satisfies the separation of hold-out proteins and ensures no shared binding partners between the training and test sets, i.e. the three types of data leakage issues mentioned above are eliminated (see Supplementary Section S1.2 for dataset splitting details).

We further check the sequence and structural similarities between held-out CATH test set and training set using BLASTP (Altschul *et al.* 1997) and TM-align (Zhang and Skolnick 2005), respectively, to confirm that there is no sequence-level and structure-level data leakage. Ultimately, in the held-out CATH test set, the sequence identity is below 30% with criteria as follows: (query coverage≥80% OR subject coverage≥80%), and none of the mutation entries are easy mutations (maximum TM-score ≥0.6). Notably, in this work, a mutation is defined as hard if its maximum TM-score <0.6 when compared to the training set, and as easy otherwise.

Here, we introduce a new way of data splitting with non-leaking of CATH superfamily, i.e. we devise a greedy strategy for data splitting with criteria as follows: proportion of CATH superfamilies in test set ≥30%. After that, we obtain an independent blind test set, i.e. held-out CATH test set, which contains 54 CATH superfamilies, 53 PPIs and 813 mutations, i.e. 30.17% of CATH superfamilies, 15.45% of PPIs and 12.12% of mutations compared to SKEMPI v2.0, as shown in Fig. 1.

##  

### 2.2 Overview of CATH-ddG

**Figure 2.**
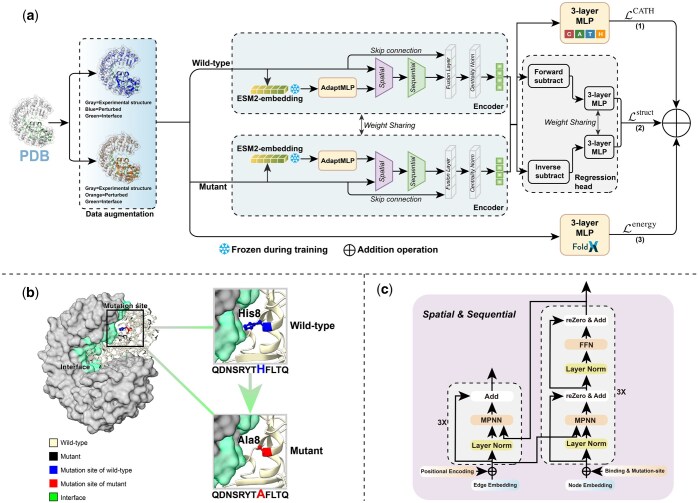
(a) Overview of the proposed CATH-ddG framework. The framework is composed of three task branches: (1) self-supervised learning, (2) structure-based supervised learning, and (3) energy-based supervised learning task. To utilize the advantage of PLMs to capture complex mapping relationships between sequence and function, we also fine-tune ESM2 via adapter with a simple nonlinearity AdaptMLP. (b) An overview of mutational microenvironment in protein complex (PDB ID: 1A4Y), which is defined as the *k*-nearest neighbor graph to the mutation site and interface. (c) The architecture of spatial and sequential geometric encoder with optimized ProteinMPNN as the backbone.

Here, we present a novel framework, CATH-ddG, which consists of three components for ΔΔG prediction (Fig. 2a). First, a self-supervised learning task enhances representations through multi-label classifier of CATH class categories. Second, a Siamese architecture geometric encoder employs shared weights, which consists of a spatial and sequential encoder with optimized ProteinMPNN (Dauparas *et al.* 2022) as the backbone, followed by the fusion layer and residue CentralityNorm layer. Third, the energy-based component adopts a lightweight module that combines FoldX-derived energy terms to provide the differences in energy change between wild-type and mutant.

### 2.3 Definition of mutational microenvironment

The mutational microenvironment of the mutation sites describes their surrounding sequence and structural contexts (Fig. 2b). Given a protein complex structure, we introduce a way whereby the mutational microenvironment is constructed as a cropped patch. First, the mutation site M and protein–protein interface I are selected as an anchor residue set of the protein complex, and then we calculate the Euclidean distances between any other residues in the complex and the anchor residue set relative to residue centroid $C_{\beta }$ atoms. Notably, the missing $C_{\beta }$ is updated with virtual $C_{\beta }$ using the same algorithm as ProteinMPNN. Here, residues with solvent accessible surface area difference >$1\;{Å}^{2}$ from the complex to the single chain are considered as an interface. Finally, we select the 256 nearest neighbor residues to the anchor residue set to represent the mutational microenvironment, and define it as the input patch.

Within the input patch residues, we use three kinds of edges to construct *k*-nearest neighbor graph for each residue to represent different interactions between these residues, and we have validated the selection of hyper-parameters $k=\{k_{1},k_{2},k_{3}\}$ in our previous work (Yu *et al.* 2024).

1. Radial edge with distance $k_{1}$. Each residue is connected to its $k_{1}=20$ nearest neighbors with respect to the Euclidean distances of $C_{\beta }$ atoms.
2. Long-range edge with distance $k_{2}$. To capture those interactions that are long-range in sequence but local in Euclidean space, neighbors of top $k_{2}=7$ residues are linked according to their ascending order of Euclidean distances, and discarded if their sequence distances are not greater than $k_{3}=2$.
3. Sequential edge with distance $k_{3}$. For two residues with a sequence distance lower than $k_{3}=2$, we add a sequential edge between them, the position of which is determined by their relative positions within the protein chain.

Formally, we define the microenvironment of each mutational anchor residue set {M,I} as three subgraphs $\{G_{i}^{\left(k_{1}\right)},G_{i}^{\left(k_{2}\right)},G_{i}^{\left(k_{3}\right)}\}\subseteq G$ of the protein complex graph G, with its node set $\left\{V_{G_{i}^{\left(k_{1}\right)}},V_{G_{i}^{\left(k_{2}\right)}},V_{G_{i}^{\left(k_{3}\right)}}\}\subseteq \{M,I\right\}$ defined as follows:

(1) $$V_{G_{i}^{\left(k_{1}\right)}}=\{v_{j}|(d_{ij},\leq ),|d_{ij}|=k_{1}\},\;$$

 

(2) $$V_{G_{i}^{\left(k_{2}\right)}}=\{v_{j}|(d_{ij},\leq ),|d_{ij}|=k_{2},|j-i|\geq k_{3}\},\;$$

 

(3) $$V_{G_{i}^{\left(k_{3}\right)}}=\{v_{j}||j-i|<k_{3}\},\;$$

where $d_{ij}$ denotes the Euclidean distance between $v_{i}$ and $v_{j}$, $v_{j}\in N_{\left(i,k\right)}$, $N_{\left(i,k\right)}$ is the neighborhood of residue $v_{i}\in \{M,I\}$ in the *k*-nearest graph of spatial space and sequential space, $k_{1},k_{2}$, and $k_{3}$ are the radial distance, long-range distance, and sequence distance, respectively, and $x_{i}$ and $x_{j}$ are the 3D coordinates of $C_{\beta }$ atoms for node $v_{i}$ and $v_{j}$, respectively.

### 2.4 Spatial and sequential geometric encoder

In spatial geometric encoder, we iterate through the residues of the input patch to select 3D spatial cropping according to the $k_{1}$ and $k_{2}$ constraints. Thus, the spatial graph consists of $k_{1}+k_{2}$ edges for each residue. In sequential geometric encoder, we iterate to select contiguous cropping according to the $k_{3}$ constraint, resulting in the sequential graph consisting of $k_{3}$ edges for each residue. Notably, we have added an index gap of 100 between chains, which ensures that we obtain the correct contiguous sequential cropping on the boundary for each protein chain.

The sequential geometric encoder has the same architecture with spatial geometric encoder. As shown in Fig. 2c, the overall geometric structure encoder forward process at *l*th layer is defined as:

(4) $$m_{ij}^{\left(l\right)}=\mathrm{MLP}(\mathrm{Cat}(\mathrm{LN}(h_{i}^{\left(l-1\right)}),\mathrm{LN}(h_{j}^{\left(l-1\right)}),e_{ij}^{\left(l-1\right)})),$$

 

(5) $$h_{i}^{\left(l\right)}=h_{i}^{\left(l-1\right)}+M^{\left(l\right)}⊙\left(\alpha^{\left(l\right)}\frac{\sum_{j\in N_{\left(i,k\right)}}m_{ij}^{\left(l\right)}}{\lambda }\right),$$

 

(6) $$h_{i}^{\left(l\right)}=h_{i}^{\left(l\right)}+M^{\left(l\right)}⊙(\alpha^{\left(l\right)}\mathrm{FFN}(\mathrm{LN}(h_{i}^{\left(l\right)}))),$$

 

(7) $$e_{ij}^{\left(l\right)}=\mathrm{MLP}(\mathrm{Cat}(h_{i}^{\left(l\right)},h_{j}^{\left(l\right)},\mathrm{LN}(e_{ij}^{\left(l-1\right)}))),$$

 

(8) $$e_{ij}^{\left(l\right)}=e_{ij}^{\left(l-1\right)}+M^{\left(l\right)}⊙(e_{ij}^{\left(l\right)}),$$

where MLP(·) is multi-layer perceptron with ReLU(·) (Glorot *et al.* 2011) as the activation function, LN(·) indicates layer normalization without bias (Xu *et al.* 2019), $N_{\left(i,k\right)}$ denotes the k-nearest neighbors of node $v_{i}$, FFN(·) is a feed-forward network, $\alpha^{\left(l\right)}$ is learnable scalar for reZero (Bachlechner *et al.* 2021), λ=30, and the dropout mask vector $M^{\left(l\right)}∼\text{Bernoulli}(p=0.1)$. Notably, the node inputs of spatial and sequential geometric encoders are $h_{i}^{\left(0\right)}$ and $h_{i}^{\text{spatial}}$, respectively, i.e. the node inputs of sequential encoder are the outputs of the spatial encoder. Finally, we take the output $h_{i}^{\text{spatial}}$ and $h_{i}^{\text{sequential}}$ as node representations, and $e_{ij}^{\text{spatial}}$ and $e_{ij}^{\text{sequential}}$ as edge representations, respectively, which are used as inputs to the fusion layer. Subsequently, the fusion layer is given by:

(9) $$h_{i}^{\text{enc}}=\mathrm{Cat}(h_{i}^{\text{spatial}},h_{i}^{\text{sequential}})W,\;e_{ij}^{\text{enc}}=\mathrm{Merge}(e_{ij}^{\text{spatial}},e_{ij}^{\text{sequential}}),$$

 

(10) $$m_{ij}=\mathrm{MLP}(\mathrm{Cat}(h_{i}^{\left(0\right)},h_{j}^{\left(0\right)},h_{i}^{\text{enc}},h_{j}^{\text{enc}},e_{ij}^{\text{enc}})),$$

 

(11) $$h_{i}^{\text{fusion}}=\mathrm{LN}\left(h_{i}^{\text{enc}}+M⊙\left(\frac{\sum_{j\in N_{\left(i,k\right)}}m_{ij}}{\lambda }\right)\right),$$

where Cat(·) denotes concatenation operation across dimensions, Merge(·) represents merge operation across edge channels, and $W\in R^{2d\times d}$ is the trainable parameters for node fusion transformation. Taking the node outputs of the fusion layer, which are normalized by the residue CentralityNorm layer, leads to the representations $h_{i}$ of CATH-ddG as:

(12) $$\hat{c_{i}}=\frac{\sum_{j\in N_{\left(i,k\right)}}c_{ij}+w}{{\left\|\sum_{j\in N_{\left(i,k\right)}}c_{ij}+w\right\|}_{2}},$$

 

(13) $$h_{i}=\hat{c_{i}}\cdot h_{i}^{\text{fusion}},$$

where $\hat{c_{i}}\in [0,1]$ is $l_{2}$ normalization for residue $r_{i}$, $c_{ij}$ is the residue centrality of *k*-nearest neighbor of $r_{i}$ defined in McPartlon and Xu (2023), *w* is a learnable scalar parameter, $i\in \{L\}\overset{\Delta }{=}\{1,2,\ldots ,L\}$, L=256 is the length of cropped residue patch.

### 2.5 Training loss and inference

#### 2.5.1 Structure-based loss

From the thermodynamic perspective, the change in binding affinity caused by amino acid substitution is calculated as follows:

(14) $$\Delta \Delta G=\Delta G_{\text{mt}}-\Delta G_{\text{wt}},$$

where $\Delta G_{\text{mt}}$ and $\Delta G_{\text{wt}}$ are binding free energies of mutant and wild-type, respectively. Here, in line with Equation (14), ΔΔG is predicted by applying three-layer MLP(·) on the CATH-ddG representations h(·) as follows:

(15) $$\Delta \Delta G_{\text{wt}\to \text{mt}}=\mathrm{MLP}(\max(h(MT_{\hat{\chi }}))-\max(h(WT_{\hat{\chi }}))),$$

where max(·) denotes max-pooling operation after masking non-mutated sites, $WT_{\hat{\chi }}$ and $MT_{\hat{\chi }}$ indicate the features of wild-type and mutant where the side-chain dihedral angles $\hat{\chi }$ of mutation sites are masked, respectively. According to the thermodynamics rule at equilibrium, ΔΔG should satisfy the antisymmetric property, i.e. $\Delta \Delta G_{\text{wt}\to \text{mt}}=-\Delta \Delta G_{\text{mt}\to \text{wt}}$. Following the mean squared error (MSE) loss, the definition of the loss function for the structure predictor is:

(16) $$L^{\text{struct}}=\frac{\mathrm{MSE}(\Delta \Delta G_{\text{wt}\to \text{mt}}^{\text{struct}},\Delta \Delta G)+\mathrm{MSE}(\Delta \Delta G_{\text{mt}\to \text{wt}}^{\text{struct}},-\Delta \Delta G)}{2}.$$

Structure-based models are superior at capturing interactions within proteins and with other biomolecules, which typically leads to better predictions of mutation effects, especially for proteins with significant conformational changes. For instance, Wang *et al.* (2023) found that the performance of multiple mutations is better than that of single mutations due to the evolutionary constraints of mutations, which results in more stable 3D structures. However, Cai *et al.* (2024) revealed that structure-based approaches tend to have low correlations when modeling weak interactions. To investigate how to mitigate this deficiency, we perform structure alignment between wild-type and mutant proteins on SKEMPI v2.0 and held-out CATH test set by using align module of PyMOL (DeLano 2002), which carries out sequence alignment followed by structural superposition and works well on proteins with high sequence similarity (identity ≥30%).

**Figure 3.**
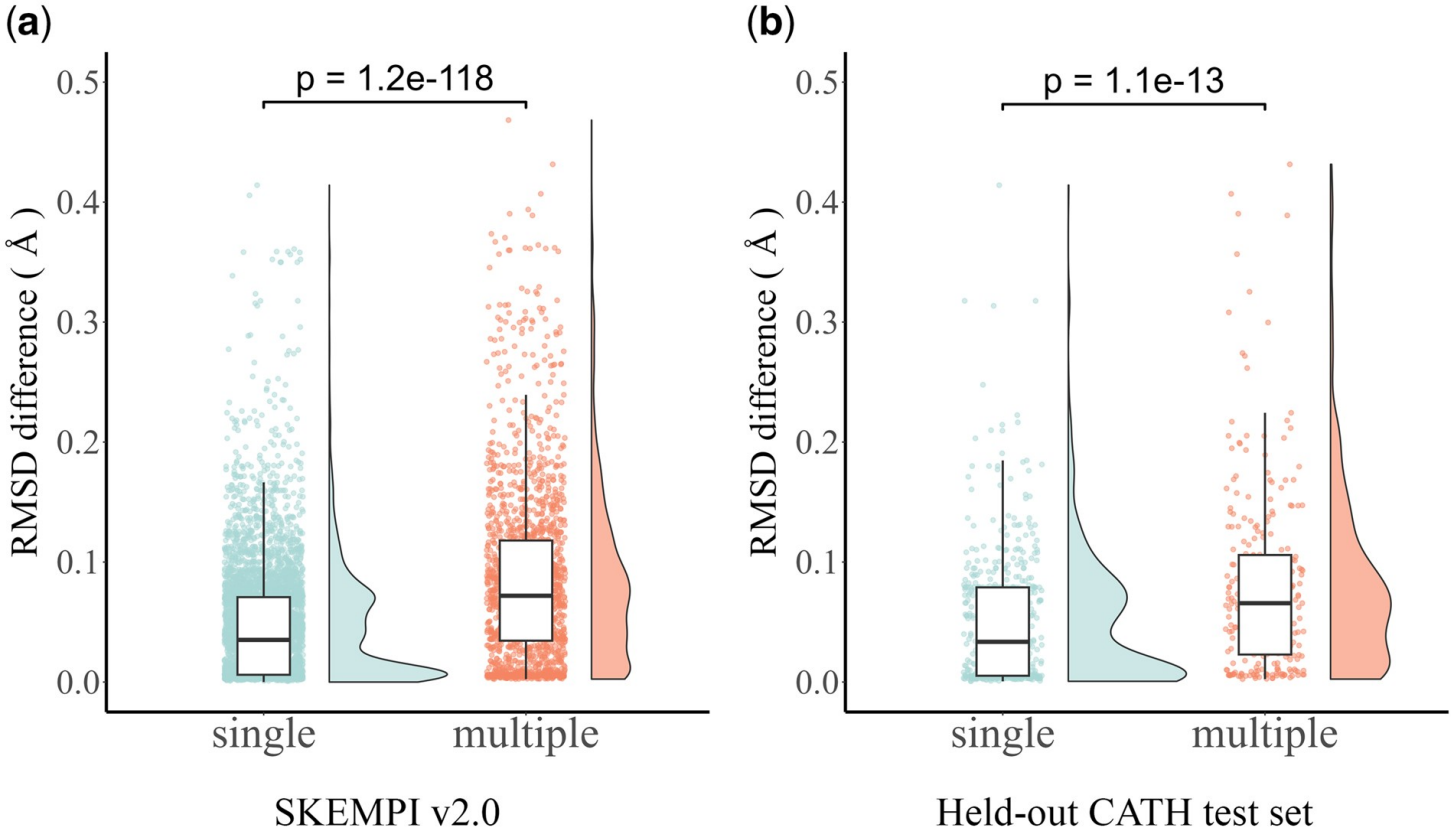
The RMSD differences between wild-type and mutant for single and multiple mutations on SKEMPI v2.0 dataset (a) and held-out CATH test set curated from SKEMPI v2.0 (b).

As shown in Fig. 3, the median differences in root mean square deviation (RMSD) between wild-type and mutant structures for single mutations are significantly lower than that of multiple mutations, which indicates that the better performance of multiple mutations may arise from differences in conformational changes. These findings fundamentally reveal the relationship between single and multiple mutations, and advance our understanding on binding-induced folding in PPIs. Thus, we modify the $L^{\text{struct}}$ to consider the difference in convergence rate between single and multiple mutations during training with stochastic gradient descent, which is defined as follows:

(17) $$L^{\text{struct}}=\beta \cdot L_{\text{single}}^{\text{struct}}+(1-\beta )\cdot L_{\text{multiple}}^{\text{struct}},$$

where $L_{\text{single}}^{\text{struct}}$ and $L_{\text{multiple}}^{\text{struct}}$ are the mean loss of single and multiple mutation entries across the mini-batch, respectively, and we set weight hyper-parameter β=0.6 in our experiments (see Supplementary Section S1.3 for different settings of β).

#### 2.5.2 Energy-based loss

Moreover, the second training loss $L^{\text{energy}}$ is to leverage the physics-based energy prior knowledge regarding the interaction energy difference between the wild-type and mutant structure, which is modeled as a three-layer MLP(·). Then, $L^{\text{energy}}$ is calculated by utilizing MSE loss, including forward predicted $\Delta \Delta G_{\text{wt}\to \text{mt}}^{\text{energy}}$ and its ground truth, as well as reverse $\Delta \Delta G_{\text{mt}\to \text{wt}}^{\text{energy}}$ for antisymmetric property:

(18) $$\Delta \Delta G_{\text{wt}\to \text{mt}}^{\text{energy}}=\mathrm{MLP}(\mathrm{Inte}r_{\text{mt}}-\mathrm{Inte}r_{\text{wt}}),$$

 

(19) $$\Delta \Delta G_{\text{mt}\to \text{wt}}^{\text{energy}}=\mathrm{MLP}(\mathrm{Inte}r_{\text{wt}}-\mathrm{Inte}r_{\text{mt}}),$$

 

(20) $$L^{\text{energy}}=\frac{\mathrm{MSE}(\Delta \Delta G_{\text{wt}\to \text{mt}}^{\text{energy}},\Delta \Delta G)+\mathrm{MSE}(\Delta \Delta G_{\text{mt}\to \text{wt}}^{\text{energy}},-\Delta \Delta G)}{2},$$

where $\mathrm{Inte}r_{\text{wt}}$ and $\mathrm{Inte}r_{\text{mt}}$ are Interaction Energy terms generated by FoldX v5.0 from wild-type and mutant structures, respectively (see Supplementary Section S1.4 for details).

#### 2.5.3 CATH domain loss

To integrate protein domain information for better representation learning, we incorporate the CATH domain constraint as a multi-label classification self-supervised learning task. Specifically, the prediction of CATH superfamilies is employed only as an auxiliary task to enhance the overall representations during training. Thus, the third training loss $L^{\text{CATH}}$ is the binary cross-entropy (BCE) loss function between the predicted probabilities (${\hat{y}}^{\text{CATH}}$) and their ground truth associated labels:

(21) $$L^{\text{CATH}}=\frac{\mathrm{BCE}({\hat{y}}_{\text{wt}}^{\text{CATH}},y_{\text{wt}}^{\text{CATH}})+\mathrm{BCE}({\hat{y}}_{\text{mt}}^{\text{CATH}},y_{\text{mt}}^{\text{CATH}})}{2}.$$

The ground truth label set $y^{\text{CATH}}$ = {0, 1, 2, 3, 4, 6} $\in R^{6}$ represents the class categories of CATH contained in protein complex, where the label 0 indicates an unannotated chain. Each complex G is associated with a subset $\subseteq y^{\text{CATH}}$, typically represented by a binary vector $y=[y_{1},\ldots ,y_{6}]$, where $y_{i}=1$ if and only if the *i*th label is associated with complex G, and 0 otherwise, see Supplementary Section S1.5 for visualization of domain distributions on CATH v4.3 and SKEMPI v2.0.

Finally, the overall loss function L is weighted by the above three training objective losses, and is defined as follows:

(22) $$L≜\gamma \cdot L^{\text{CATH}}+L^{\text{struct}}+L^{\text{energy}},$$

where we set weight hyper-parameter γ=0.5 for self-supervised learning auxiliary task in our experiments (see Supplementary Section S1.6 for different settings of γ).

#### 2.5.4 Inference

Here, we adopt an equally weighted predictor for ΔΔG of CATH-ddG (see Supplementary Section S1.7 for prediction results under different weight settings), defined as follows:

(23) $$\Delta \Delta G_{\text{wt}\to \text{mt}}=0.5\cdot \Delta \Delta G_{\text{wt}\to \text{mt}}^{\text{struct}}+0.5\cdot \Delta \Delta G_{\text{wt}\to \text{mt}}^{\text{energy}}.$$

### 2.6 Node features and edge features

#### 2.6.1 Node features

Residue distance features $f_{i}^{\text{RBF}}$ of $r_{i}$ are represented by eight Gaussian radial basis functions (RBFs) equally spaced from 0.2 to 6.2 Å between atoms in backbone atom set $\mathrm{BB}=\{N,C_{\alpha },C,O,C_{\beta }\}$ and side-chain atomic set SC. We fine-tune ESM2 embeddings $h^{\text{ESM}2}(r_{i})$ by freezing the 650M version of ESM2 and assembling adapter layer via AdaptMLP(·), which is denoted as $f_{i}^{\text{ESM}2}$. Dihedral angles $\left\{\phi_{i},\psi_{i},\chi_{i}^{\left(1\right)},\chi_{i}^{\left(2\right)},\chi_{i}^{\left(3\right)},\chi_{i}^{\left(4\right)}\right\}$ of residue $r_{i}$ are masked when missing and embedded into the torus space, denoted as $f_{i}^{\text{dihedral}}$, where each angle ranges from $\left(-180^{{}^{\circ}},180^{{}^{\circ}}\right]$. We follow Jin *et al.* (2022) to build the physico-chemical characteristic for residue $r_{i}$, which consists of six features and is denoted as $f_{i}^{\text{physico–chemical}}$. The one_hot embeddings $f_{i}^{\text{binding–partner}}$ and $f_{i}^{\text{mutation–site}}$ indicate whether residue $r_{i}$ is on the binding partner or mutation site, respectively. Collectively, we term the composition of above blocks as the node features:

(24) $$\begin{array}{cc} f_{i}^{\text{RBF}} & =\overset{2}{\underset{t=1}{\mathrm{Cat}}}(\mathrm{RBF}(||x_{\left(i,m_{t}\right)}-x_{\left(i,n_{t}\right)}||)), \end{array}$$

 

(25) $$\begin{array}{cc} f_{i}^{\text{ESM}2} & =\mathrm{AdaptMLP}(h^{\text{ESM}2}(r_{i})), \end{array}$$

 

(26) $$\begin{array}{cc} f_{i}^{\text{dihedral}} & =\{\;\text{sin}\;,\;\text{cos}\;\}\times \{\phi_{i},\psi_{i},\chi_{i}^{\left(1\right)},\chi_{i}^{\left(2\right)},\chi_{i}^{\left(3\right)},\chi_{i}^{\left(4\right)}\}, \end{array}$$

 

(27) $$\begin{array}{cc} h_{i}^{\left(0\right)} & =\mathrm{MLP}(\mathrm{Cat}(f_{i}^{\text{RBF}},f_{i}^{\text{ESM}2},f_{i}^{\text{dihedral}})), \end{array}$$

 

$$h_{i}^{\left(0\right)}=h_{i}^{\left(0\right)}+\mathrm{MLP}(f_{i}^{\text{physico–chemical}})+\mathrm{Linear}(f_{i}^{\text{binding–partner}})$$

 

(28) $$+\mathrm{Linear}(f_{i}^{\text{mutation–site}}),$$

where $x_{\left(i,m_{t}\right)}$, $x_{\left(i,n_{t}\right)}$ are the 3D coordinates of atom $m_{t}$ and $n_{t}$ for residue $r_{i}$, and $\left(m_{t},n_{t})\in \{BB_{\left(i\right)}\times BB_{\left(i\right)}\cup SC_{\left(i\right)}\times SC_{\left(i\right)}\right\}$. Ultimately, $h_{i}^{\left(0\right)}$ is the input node features for residue $r_{i}$.

#### 2.6.2 Edge features

The edge features consist of the following five parts:

(29) $$e_{ij}=\mathrm{Cat}(e_{ij}^{\text{distance}},e_{ij}^{\;\text{direction}},e_{ij}^{\text{orientation}},e_{ij}^{\;\text{position}},e_{ij}^{\text{aa}\_\text{pair}}).$$

We add edge features with 16 RBFs equally spaced from 2.0 to 22.0 Å for distances between atoms of residue $r_{i}$ and its neighbors $r_{j}$, defined as follows:

(30) $$e_{ij}^{\text{distance}}=\overset{3}{\underset{t=1}{\mathrm{Cat}}}(\mathrm{RBF}(||x_{\left(i,m_{t}\right)}-x_{\left(j,n_{t}\right)}||)),$$

where $x_{\left(i,m_{t}\right)}$, $x_{\left(j,n_{t}\right)}$ are the 3D coordinates of atom $m_{t}$ and $n_{t}$ for residue $r_{i}$ and $r_{j}$, respectively, $\left(m_{t},n_{t})\in \{BB_{\left(i\right)}\times BB_{\left(j\right)}\cup C_{\beta ,(i)}\times SC_{\left(j\right)}\cup SC_{\left(i\right)}\times C_{\beta ,(j)}\right\}$. The computational algorithms for $e_{ij}^{\text{direction}}$ and $e_{ij}^{\text{orientation}}$ are the same as ProteinMPNN.

Relative positional features $f_{ij}^{\text{relative–pos}}$ are encoded to infer the positions of residues, which are clipped between $\left[-r_{\text{max}},r_{\text{max}}\right]$, with $r_{\text{max}}=32$ defined in AlphaFold-Multimer (Evans *et al.* 2021). Furthermore, we provide $f_{ij}^{\text{same–chain}}$ to encoding whether the residues are from the same chains. In addition, pairwise correlation between residues has been shown to be a useful feature in predicting contacts and 3D structure (Evans *et al.* 2021). Here, the amino acid type $r_{i}$ is in set $\{0,1,\ldots ,20\}\in R^{21}$ with $r_{\text{type}}$ dimensions for an additional unknown residue type, and residue pair embeddings $f_{ij}^{\text{aa}\_\text{pair}}\in R^{21\times 21}$
 

(31) $$d_{ij}=\mathrm{clip}(f_{i}^{\text{residue–index}}-f_{j}^{\text{residue–index}}+r_{\text{max}},0,2\cdot r_{\text{max}}),$$

 

(32) $$f_{ij}^{\text{relative–pos}}=\mathrm{one}\_\mathrm{hot}(d_{ij},2\cdot r_{\text{max}}+1),$$

 

(33) $${\text{chain}}_{ij}=\mathrm{clip}(|f_{i}^{\text{chain–id}}-f_{j}^{\text{chain–id}}|,0,1),$$

 

(34) $$f_{ij}^{\text{same-chain}}=\mathrm{one}\_\mathrm{hot}({\text{chain}}_{ij},2),$$

 

(35) $$e_{ij}^{\text{position}}=\mathrm{Linear}(f_{ij}^{\text{relative–pos}})+\mathrm{Linear}(f_{ij}^{\text{same–chain}}),$$

 

(36) $$f_{ij}^{\text{aa}\_\text{pair}}=r_{i}\cdot r_{\text{type}}+r_{j},$$

 

(37) $$e_{ij}^{\text{aa}\_\text{pair}}=\mathrm{Linear}(f_{ij}^{\text{aa}\_\text{pair}}).$$

### 2.7 Data augmentation

Data augmentation is a widely used training technique that can greatly scale up the amount and diversity of data without collecting new samples. In this article, we leverage data augmentation from three different perspectives.

Firstly, training with noise has been proven to be a valid training strategy for protein design (Dauparas *et al.* 2022) and coordinate denoising in 3D molecular pre-training (Feng *et al.* 2023). Here, we propose a novel hybrid noise strategy, i.e. imposing two-step Gaussian noise with different variances on the atomic coordinates of rigid backbone BB and flexible side-chain SC (see Supplementary Section S1.8 for visualization). Specifically, the first step is to perturb the rigid and flexible components with variances $\delta^{2}={\left(0.15\;Å\right)}^{2}$ and ${\left(0.25\;Å\right)}^{2}$, respectively, which leads to the perturbed dihedral features of the nodes. The second step involves perturbing the original atomic coordinates again with the same Gaussian noise implemented in the first step, which yields the perturbed distance, direction, and orientation features of the nodes and edges. In this way, our hybrid noise strategy provides reasonable anisotropic conformational distribution constraints for proteins.

Secondly, side-chain dihedral angles of certain amino acids, including $\chi^{\left(2\right)}$ of aspartic acid (ASP), $\chi^{\left(3\right)}$ of glutamic acid (GLU), $\chi^{\left(2\right)}$ of phenylalanine (PHE), and $\chi^{\left(2\right)}$ of tyrosine (TYR), exhibit symmetric invariance (McPartlon and Xu 2023), which means that flipping the corresponding dihedral angles does not affect the overall 3D structure. To this end, we utilize this property as a data augmentation strategy to generate more diversity. In particular, we rotate the side-chain dihedral angles $180^{{}^{\circ}}$ uniformly at random with probability P=.2 (see Supplementary Section S1.9 for different settings of *P*).

Finally, the chains of protein complexes are randomly shuffled to avoid bias during training.

### 2.8 Training strategy

To prevent the model from overfitting, we use an early stopping strategy with patience steps=10, and ϵ=0.02 for 150 epochs at most in our scenario. Specifically, for each epoch, we record the moving average loss calculated on the training data for every mini-batch, and we stop training if the moving average loss on the training set does not decrease (ϵ=0.02) within 10 consecutive records after 100 epochs. The batch size is set to 32 and all experiments are run on a single A100 GPU.

Scheduler with restarts are widely used to rescue the optimizer from getting stuck in saddle points and local minima. Thus, we adopt the Adam optimizer with weight decay regularization, and cyclical cosine annealing strategy with warmup restarts for the learning rate (LR) η akin to that of Loshchilov and Hutter (2017) as follows:

(38) $$\eta =\{\begin{array}{cc} \frac{T_{\text{cur}}}{T_{\text{warmup}}}\cdot \eta_{\text{max}\;},\;\;\;\;\;\;\;\;\;\;\text{if}\;\;\;\;T_{\text{cur}}\leq T_{\text{warmup}} & \\ \eta_{\text{min}}+\frac{1}{2}(\eta_{\text{max}}-\eta_{\text{min}})\left(1+\text{cos}\;\left(\frac{T_{\text{cur}}-T_{\text{warmup}}}{T-T_{\text{warmup}}}\pi \right)\right),\text{else} & \end{array}$$

where $T_{\text{warmup}}$ and *T* are the warmup and cyclical mini-batch iteration steps, respectively. $\eta_{\text{min}}$ and $\eta_{\text{max}}$ are ranges of LR, and $T_{\text{cur}}$ is the current iteration step since last restart. $\eta =\eta_{\text{max}}$ when $T_{\text{cur}}=T_{\text{warmup}}$, and $\eta =\eta_{\text{min}}$ when $T_{\text{cur}}=T$. To further bridge the differences in convergence rates derived from multi-source data, different LR bound settings, and corresponding iteration steps, the weight decays are shown in Table 1.

**Table 1.** Hyper-parameter settings for training.

	$\eta_{\text{min}}$	$\eta_{\text{max}}$	Weight decay	$T_{\text{warmup}}$	*T*
${\text{LR}}_{\text{base}}$^a^	$2.0\times 10^{-4}$	$1.0\times 10^{-3}$	$1.0\times 10^{-4}$	$4.0\times 10^{3}$	$5.0\times 10^{3}$
${\text{LR}}_{\text{FoldX}}$^b^	$2.0\times 10^{-5}$	$1.0\times 10^{-4}$	$1.0\times 10^{-6}$	$4.0\times 10^{3}$	$5.0\times 10^{3}$
${\text{LR}}_{\text{ESM}2}$^c^	$2.0\times 10^{-6}$	$1.0\times 10^{-5}$	$1.0\times 10^{-6}$	$4.0\times 10^{3}$	$5.0\times 10^{3}$

a ${\text{LR}}_{\text{base}}$ is the base LR of CATH-ddG.

b ${\text{LR}}_{\text{FoldX}}$ is the LR of FoldX MLP predictor.

c ${\text{LR}}_{\text{ESM}2}$ is the LR of ESM2 adapter layer AdaptMLP.

## 3 Results

### 3.1 Experimental setup

Following RDE-Network, the performance of CATH-ddG is evaluated using five metrics: Pearson correlation coefficient (PearsonR), spearman’s rank correlation coefficient (SpearmanR), minimized root mean squared error (RMSE), minimized mean absolute error (MAE), and AUROC with respect to the sign of ΔΔG values. In addition, the correlation of specific PPI is of greater interest in practice. Therefore, we group mutations by PPIs and filter out less than 10 mutations, which yields two additional metrics: average per-PPI PearsonR and average per-PPI SpearmanR.

To compare with the baselines (details in Supplementary Section S1.10) on the SKEMPI v2.0 dataset, we use three different data splitting strategies: the held-out CATH blind set proposed in this work (Table 2), the held-out protein blind test according to PPIFORMER (Table 3), and three-fold cross-validation under the randomized PPIs splitting based on RDE-Network (Supplementary Table S2). The baseline models presented in Table 2 are retrained and retested using their default parameter settings. The results of the baseline models shown in Table 3 and Supplementary Table S2 are cited from PPIFORMER and Prompt-DDG, respectively. Additionally, the case study results, presented in Tables 4 and 5, focus on optimizing antibody variants targeting HER2 and variants in the RBD of SARS-CoV-2 binding to ACE2. The best values for each metric in all tables are highlighted in bold.

**Table 2.** Blind test performance under single, multiple, and overall mutations on held-out CATH test set curated from SKEMPI v2.0 for benchmarking.

Input	Category	Method	Mutations	Overall ^a^					per-PPI ^a^		Reference
				PearsonR↑	SpearmanR↑	RMSE↓	MAE↓	AUROC↑	PearsonR↑	SpearmanR↑	
Structure-only	Energy-based	FoldX	All	0.4603	0.5246	2.3083	1.6837	0.7541	0.4932	0.4303	Delgado *et al.* (2019)
			Single	0.4507	0.4919	1.7453	1.3017	0.7125	0.5001	0.4458	
			Multiple	0.4029	0.3698	3.1460	2.4289	**0.8353**	0.5831	0.5479	
		flex ddG	All	0.6081	0.6096	2.0643	**1.4946**	0.7637	0.5083	0.4542	Barlow *et al.* (2018)
			Single	0.5697	0.5330	1.6068	1.2002	0.7073	0.5681	0.4633	
			Multiple	**0.6198**	**0.5616**	**2.6975**	**2.0545**	0.8278	**0.6161**	**0.6399**	
	Pre-training based	ProteinMPNN	All	0.1837	0.3668	2.5559	1.8612	0.6788	0.1878	0.2543	Dauparas *et al.* (2022)
			Single	0.3354	0.3194	1.8419	1.4501	0.7191	0.2504	0.2740	
			Multiple	0.0116	0.3088	3.4371	2.5612	0.5616	0.1868	0.1263	
		ESM-IF	All	0.1421	−0.1813	2.5738	1.9376	0.3916	−0.0876	−0.1331	Hsu *et al.* (2022)
			Single	−0.1965	−0.2351	1.9171	1.4784	0.3300	−0.2250	−0.2310	
			Multiple	0.3206	−0.0940	3.2558	2.5006	0.5533	0.0351	0.0527	
Sequence & structure	Pre-training based	RDE-Network	All	0.4588	0.4703	2.3103	1.7029	0.7452	0.3500	0.2880	Luo *et al.* (2023)
			Single	0.3830	0.3756	1.8061	1.3547	0.6899	0.3465	0.2534	
			Multiple	0.4498	0.3789	3.0700	2.4025	0.8196	0.5032	0.4729	
		DiffAffinity	All	0.3144	0.3626	2.4684	1.7393	0.6251	0.3233	0.2494	Liu *et al.* (2023)
			Single	0.2639	0.2927	1.8859	1.3838	0.5940	0.3358	0.2500	
			Multiple	0.2458	0.2602	3.3318	2.4426	0.6729	0.2903	0.2882	
		PPIFORMER	All	0.2654	0.3145	2.5069	1.8191	0.6740	0.2169	0.2164	Bushuiev *et al.* (2024)
			Single	0.2090	0.2558	1.9120	1.4526	0.6675	0.2694	0.2549	
			Multiple	0.2197	0.1910	3.3533	2.4838	0.6610	0.4238	0.4238	
		Prompt-DDG	All	0.3538	0.397	2.4320	1.7556	0.6851	0.3212	0.3025	Wu *et al.* (2024)
			Single	0.2779	0.3139	1.8781	1.3889	0.6217	0.3053	0.2842	
			Multiple	0.3240	0.2618	3.2518	2.4907	0.7891	0.4812	0.4404	
	Supervised learning	DDAffinity	All	0.3984	0.4026	2.3849	1.7871	0.7248	0.2310	0.1956	Yu *et al.* (2024)
			Single	0.3497	0.3381	1.8317	1.3996	0.6846	0.2529	0.1846	
			Multiple	0.3803	0.3465	3.1790	2.4692	0.7765	0.4104	0.3427	
		CATH-ddG	All	**0.6150**	**0.6269**	**2.0504**	1.5047	**0.7805**	**0.5260**	**0.4940**	–
			Single	**0.6250**	**0.5937**	**1.5263**	**1.1377**	**0.7464**	**0.5690**	**0.5090**	
			Multiple	0.5746	0.5275	2.8131	2.2518	0.8325	0.5965	0.5507	

a Bold values indicate the best results.

**Table 3.** Blind test set performance averaged across held-out proteins under PPIFORMER data splitting.

Category	Method	PearsonR↑^c^	SpearmanR↑^c^
Energy-based	FoldX^a^	0.56	0.53
	flex ddG^b^	0.57	0.55
Sequence alignment-based	GEMME^b^	0.41	0.38
Pre-training based	MSA Transformer^b^	0.36	0.31
	ESM-IF^b^	0.18	0.18
	RDE-Network^b^	0.30	0.24
	PPIFORMER^b^	0.46	0.44
	DiffAffinity^a^	0.41	0.31
	Prompt-DDG^a^	0.13	0.12
Supervised learning	DDAffinity^a^	0.38	0.34
	CATH-ddG	**0.63**	**0.56**

a Results are from released tool or source code.

b Results are from PPIFORMER (Bushuiev *et al.* 2024).

c Bold value indicates the best result.

**Table 4.** Evaluation on the binding affinity of HER2 binders.

Category	Method	PearsonR↑^c^	SpearmanR↑^c^
Energy-based	FoldX^a^	0.246	0.329
Pre-training based	GearBind+P^a^	0.515	0.517
	GearBind+Ensemble^a^	0.579	0.608
	RDE-Network^b^	0.465	0.481
	Prompt-DDG^b^	0.413	0.432
	DiffAffinity^b^	0.590	0.524
Supervised learning	DDGPred^a^	0.387	0.388
	GearBind^a^	0.478	0.475
	DDAffinity^b^	0.386	0.356
	CATH-ddG	**0.607**	**0.639**

a Results are from GearBind (Cai *et al.* 2024).

b Results are from released source code.

c Bold value indicates the best result.

**Table 5.** Evaluation on the binding affinity of SARS-CoV-2 RBD.

Method	FoldX^a^	RDE-Network^a^	DiffAffinity^a^	DDAffinity^a^	CATH-ddG
PearsonR↑^b^	0.385	0.403	0.305	0.456	**0.579**

a Results are from DDAffinity (Yu *et al.* 2024).

b Bold value indicates the best result.

### 3.2 Comparison with state-of-the-art methods

We have conducted three experiments to assess CATH-ddG’s ability to predict ΔΔG across different data splittings on SKEMPI v2.0. For computational efficiency analysis of CATH-ddG, see Supplementary Section S1.11 for details.

The first experiment is to predict ΔΔG on held-out CATH test set. As shown in Table 2, on these challenging test PPIs, CATH-ddG significantly outperforms all deep learning baselines across all evaluation metrics, with overall PearsonR value of 0.6150 for CATH-ddG compared to 0.4588 for the SOTA method RDE-Network. Notably, this non-leaking evaluation of CATH superfamily demonstrates that traditional physics-based force fields, exemplified by flex ddG, still surpass the majority of machine learning approaches in terms of prediction performance. In contrast, CATH-ddG achieves superior or comparable predictive performance with flex ddG on all evaluation metrics. However, when it comes to speed, force field method flex ddG may not be suitable for mutational screenings as it is significantly slower by five orders of magnitude compared to machine learning methods (Bushuiev *et al.* 2024).

The second experiment is to predict ΔΔG on held-out test cases under the PPIFORMER data splitting. As shown in Table 3, CATH-ddG confidently outperforms all the baselines, including the SOTA force field method flex ddG, by achieving PearsonR of 0.63, SpearmanR of 0.56 (see Supplementary Section S1.12 for details of non-aggregated performance).

The third experiment is for a fair comparison where the data splitting setting of training and test set is the same as that of RDE-Network. As shown in Supplementary Section S1.13, CATH-ddG also achieves the best performance compared to all other baselines on all assessment metrics. Additionally, while deep learning models demonstrate superior performance on this PPI-disjoint splits, their performances decline sharply when evaluated on the PPIFORMER and CATH-ddG held-out split test sets, indicating the inherent limitations in their generalization capabilities of structure-only based methods.

### 3.3 Case study

For practical applications of identifying desirable mutations from the mutation pool, we carry out evaluations on two independent cases where the models are trained on SKEMPI v2.0 with a three-fold split, and the test performance metrics are the average of inference evaluations of these three models. These case studies demonstrate the capability of CATH-ddG to enhance the effects of mutations on 419 antibody variants targeting HER2 and 285 variants of the SARS-CoV-2 RBD binding to ACE2.

#### 3.3.1 Evaluation on the HER2 binders

The HER2 binders test set is sourced from Cai *et al.* (2024) (PDB ID: 1N8Z), which was *de novo* designed to enhance the quality and controllability of the antibody trastuzumab using generative artificial intelligence. The set includes 419 variants with *de novo* designed complementarity-determining region (CDR) loops, which have been experimentally validated via surface plasmon resonance (SPR) to establish their reliability for evaluating AI-driven antibody design.

The variants of antibody trastuzumab exhibit high edit distance, averaging 7.6, which is a challenge for ΔΔG predictions that are trained on low edit distance dataset SKEMPI v2.0 (Cai *et al.* 2024). The results in Table 4 show that CATH-ddG achieves the best performance measured by PearsonR and SpearmanR, which are 2.88% and 5.10% higher than SOTA baseline DiffAffinity and GearBind + Ensemble, respectively, indicating that it has good generalizability to different antibodies (see Supplementary Section S1.14 for details).

#### 3.3.2 Mutation effect prediction of SARS-CoV-2 RBD

To better comprehend the epistasis of mutation effects and to interpret the evolution of SARS-CoV-2, Starr *et al.* (2022) conducted deep mutational scanning to experimentally measure site-saturation mutagenesis in the ancestral Wuhan-Hu-1 RBD and its four variants (PDB ID: 6M0J). According to (Liu *et al.* 2023), 15 critical mutation sites in SARS-CoV-2 RBD have been identified, leading to a total of 285 single mutations for benchmarking. From the results reported in Table 5, CATH-ddG achieves a PearsonR value of 0.579, significantly surpassing all other baseline methods, compared to the second best PearsonR value of 0.456.

### 3.4 Ablation study

**Figure 4.**
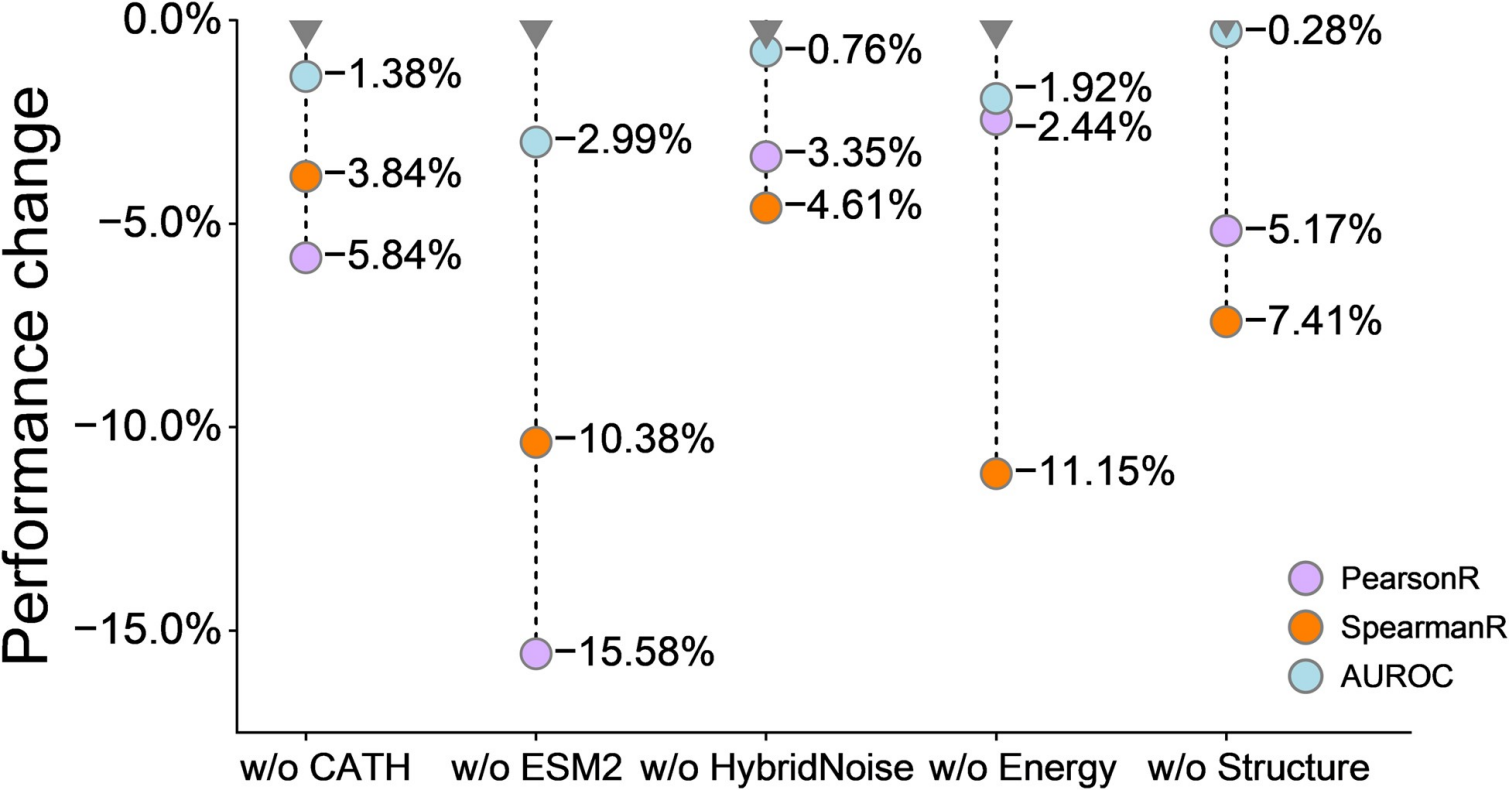
Impact of excluding individual modules on the performance metrics of the CATH-ddG model evaluated on the held-out CATH test set.

To thoroughly investigate the significance and uniqueness of each component, we have designed the following five variants of CATH-ddG: removing CATH self-supervised learning task (w/o CATH), leveraging amino acid one-hot encoding instead of ESM2 initiation (w/o ESM2), using equal variance $\delta^{2}={\left(0.20\;Å\right)}^{2}$ for backbone and side-chain atomic coordinates (w/o HybridNoise), removing FoldX energy task (w/o Energy), and removing structure ProteinMPNN-based part (w/o Structure). Through gradual deactivation of each component followed by retraining and evaluation, we observe decreases in the correlation metrics PearsonR and SpearmanR, and the accuracy metric AUROC (Fig. 4), which highlights the essential contribution of each component to the overall robustness. The experiment of w/o ESM2, with PearsonR, SpearmanR, and AUROC decreasing by 15.58%, 10.38%, and 2.99%, respectively, indicates that co-evolutionary information is crucial in determining the mutation effect on PPIs, which is pre-trained across billions of protein sequences. In addition, the experiment of w/o energy significantly reduces the SpearmanR of 11.15%, indicating the necessity of utilizing protein energetics in mutation effect on PPIs, and also suggesting that physics-based energy constraints is more helpful for ranking. The ablation studies conducted in this work are all performed on the held-out CATH test set.

## 4 Conclusion

In this article, we report a supervised deep learning architecture, CATH-ddG, which is designed to efficiently and robustly predict the effects of mutations on PPIs and enable potential applications in protein–protein binding optimization. This is of critical importance since competing methods either make comparable predictions with extremely low efficiency or perform significantly poor performance for protein complexes of unseen CATH superfamily. While protein 3D structures provide structural insights, the proposed architecture highlights the importance of integrating sequence co-evolutionary information, physics-based energy terms, and CATH homologous superfamily classification. This integrated strategy significantly enhances generalization capacity and facilitates the deep understanding of mutation effects on PPIs.

To address fair comparison with baselines, our experiments cover three different data splittings on SKEMPI v2.0. First, on blind test set of held-out CATH test set, the experiments clearly provide superior performance in correlation and accuracy when compared to pre-training methods, and comparative performance in efficiency when compared to physics-based SOTA flex ddG. Second, on blind test set of PPIFORMER, CATH-ddG achieves PearsonR of 10.53% and SpearmanR of 1.82% improvement compared to the SOTA. Third, under RDE-Network data splitting, CATH-ddG consistently outperforms baselines in terms of all comparative performance metrics. We also demonstrate the effectiveness and generalization of CATH-ddG in interpreting SARS-CoV-2 evolution and enhancing binding affinity of *de novo* designed antibodies to HER2.

Indeed, the limitations of our study highlight potential directions for future research. High-resolution protein complex crystal structures are crucial for accurate ΔΔG prediction but are often unavailable. To bridge this gap, leveraging structure prediction tools like AlphaFold3 (Abramson *et al.* 2024) from sequences is a promising approach (see Supplementary Section S1.15 for details). Additionally, using tools like FoldX to generate mutant structures is computationally expensive and limits scalability. Future work should focus on predicting ΔΔG directly from wild-type structures. In conclusion, our work establishes an efficient and robust pipeline for *in silico* antibody affinity maturation, integrated with virus evolution, to aid disease therapy and research.

## Supplementary Material

btaf228_Supplementary_Data

## Data Availability

All datasets utilized in this study are publicly available, and the data and source code are available in Github, at https://github.com/ak422/CATH-ddG.
