# Supplementary material for: CATH-ddG: towards robust mutation effect prediction on protein–protein interactions out of CATH homologous superfamily
Source: Bioinformatics. 2025 Jul 15;41(Suppl 1):i362–72. doi: 10.1093/bioinformatics/btaf228 (PMC12261453; doi:10.1093/bioinformatics/btaf228)
Supplement: btaf228_Supplementary_Data [file btaf228_supplementary_data.zip › btaf228_Supplementary_Data/Wang.103.Alt text and keywords.docx]

Figure 1. Graphical representation of the training and test sets of SKEMPI v2.0 dataset split by CATH superfamily, where each node represents a CATH superfamily.

Figure 2. Graphs on our proposed CATH-ddG framework, with subfigures labelled from a to c, illustrating model architecture, mutational microenvironment and modules of encoder.

Figure 3. Graphs comparing the RMSD differences from single vs. multiple mutations on SKEMPI v2.0 dataset and held-out CATH test set, respectively, with significant RMSD differences of multiple mutations than single mutations.

Figure 4. Graphical representation of the impact of excluding CATH, ESM2, hybrid noise, FoldX and structural modules on the performance metrics of our proposed CATH-ddG model, respectively.

Table 1. Table representation of the learning rate settings for training, including the learning rate of our proposed CATH-ddG, FoldX MLP predictor and ESM2 adapter layer (AdaptMLP), respectively.

Table 2. Table performance comparison under single, multiple, and overall mutations on held-out CATH test set curated from SKEMPI v2.0, with our proposed CATH-ddG significantly outperforming all deep learning baselines across all evaluation metrics, while energy-based flex ddG achieving higher overall performance metrics but being significantly slower compared to machine learning methods.

Table 3. Table showing performance comparison of our proposed CATH-ddG with baseline methods on held-out protein test sets under PPIFORMER split, where CATH-ddG achieving Pearson R=0.63 and Spearman R=0.56, outperforming the SOTA energy-based flex ddG method with PearsonR=0.57 and SpearmanR=0.55.

Table 4. Table illustrating the performance evaluation of HER2 binder case study, with our CATH-ddG outperforming the SOTA methods DiffAffinity and GearBind+Ensemble by 2.88% PearsonR and 5.10% SpearmanR, respectively.

Table 5. Table illustrating the performance evaluation of mutations on SARS-CoV-2 RBD, with our CATH-ddG achieving a PearsonR value of 0.579, significantly surpassing all other baseline methods.

Keywords: CATH homologous superfamily; proteinMPNN; Mutation effect prediction; Protein structure
