# Supplementary material for: CATH-ddG: towards robust mutation effect prediction on protein–protein interactions out of CATH homologous superfamily
Source: Bioinformatics. 2025 Jul 15;41(Suppl 1):i362–72. doi: 10.1093/bioinformatics/btaf228 (PMC12261453; doi:10.1093/bioinformatics/btaf228)
Supplement: btaf228_Supplementary_Data [file btaf228_supplementary_data.zip › btaf228_Supplementary_Data/Wang.103.sup.1.pdf]

CATH-ddG: towards robust mutation effect prediction on protein–protein interactions out of CATH homologous superfamily

Guanglei Yu [1,2,3], Xuehua Bi [3], Teng Ma [1,2], Yaohang Li [4] and Jianxin Wang [1,2,*]
[1]School of Computer Science and Engineering, Central South University, Changsha 410083, China
[2]Hunan Provincial Key Lab on Bioinformatics, Central South University, Changsha 410083, China
[3]College of Medical Engineering and Technology, Xinjiang Medical University, Urumqi 830017, China
[4]Department of Computer Science, Old Dominion University, Norfolk, VA 23529, United States

Contents

1. Supplementary Material 1
1.1. Mutation distributions of SKEMP2 v2.0 and held-out CATH test set 1
1.2. Algorithm for dataset splitting 1
1.3. Hyper-parameter selection of β 2
1.4. The steps for generating mutant structures . . 2
1.5. Visualization of superfamily distribution between CATH v4.3 and SKEMPI v2.0 4
1.6. Hyper-parameter selection of γ 4
1.7. Detailed prediction results under different weight settings 4
1.8. Visualization of rigid and flexible components of molecular 4
1.9. Hyper-parameter selection of p 4
1.10 Details of baseline methods 4
1.11 Computational efficiency analysis of CATH-ddG 6
1.12 Detailed results under PPIFORMER data splitting 8
1.13 Detailed results under RDE-Network data splitting 9
1.14 Detailed results on HER2 test set 9
1.15 Results of CATH-ddG with AlphaFold3 predicted structures 9

1. Supplementary Material

Section 1.1 visualizes the mutation distributions of SKEMP2 v2.0 and the held-out CATH test set. Section 1.2 describes the algorithm for dataset splitting based on CATH homologous superfamilies. Section 1.3 presents detailed comparative experiments with different hyper-parameter β settings. Section 1.4 outlines the detailed steps for generating mutant structures using FoldX. Section 1.5 visualizes the differences in protein domain distribution between CATH v4.3 and SKEMPI v2.0. Section 1.6 presents detailed comparative experiments with different hyper-parameter γ settings. Section 1.7 provides detailed prediction results under various weight settings during the inference process. Section 1.8 visualizes the rigid backbone and flexible side-chain components of molecules. Section 1.9 presents detailed comparative experiments with different hyper-parameter p settings. Section 1.10 provides details of the baseline methods. Section 1.11 analyzes the computational efficiency of CATH-ddG. Section 1.12 provides detailed comparisons of CATH-ddG with state-of-the-art $\Delta\Delta G$ predictors on the held-out protein test set under PPIFORMER data splitting of SKEMPI v2.0. Section 1.13 provides comprehensive 3-fold evaluation performance of CATH-ddG under the same data splitting of RDE-Network on SKEMPI v2.0. Section 1.14 provides detailed prediction results on the independent HER2 test set for three models trained with 3-fold cross-validation of SKEMPI v2.0. Section 1.15 presents results of CATH-ddG using AlphaFold3-predicted structures as input.

1.1. Mutation distributions of SKEMP2 v2.0 and held-out CATH test set

The statistics show that the percentage of alanine mutations is $4565/11309 = 40.37\%$ in SKEMPI v2.0, and is $878/1411 = 62.23\%$ in held-out CATH datasets. The statistics show that there are different degrees of alanine mutation bias within these two datasets. In addition, to illustrate the mutation distribution differences between the SKEMPI v2.0 dataset and the held-out CATH test set, we visualize the occurrences of the 20 amino acid mutations in Figure S1.

1.2. Algorithm for dataset splitting

To create the training and testing sets from the SKEMPI v2.0 protein complex dataset, the following steps are performed:

*jxwang@mail.csu.edu.cn

(1) Using the CATH domain database ($\texttt{Domain}_{\text{cath}}$), the protein domains present in each protein complex are identified. If a protein chain does not belong to any known CATH domains, it is labeled as 0.0.0.0 ($\texttt{Domain}_{\text{zero}}$), and Foldseek is used to retrieve the relevant CATH domain information;

(2) The CATH homologous superfamilies present in all protein complexes are considered as nodes in a CATH domain graph, where edges are added between any two CATH superfamily nodes that appear in the same protein complex;

(3) The connected components of the CATH domain graph are then sorted by the number of nodes, and the training and testing sets are divided greedily, following a 0.7:0.3 ratio based on the number of CATH homology superfamily nodes;

The pseudo-code for the proposed dataset splitting algorithm, which is based on CATH homologous superfamilies, is outlined in Algorithm 1.

1.3. Hyper-parameter selection of β

In Equation 17, we set the weight hyper-parameter of structure-based loss function $\beta = 0.6$ to balance the difference in convergence rate between single and multiple mutations in our experiments. In this section, we provide comparative experiments of β in different settings, see Table S1.

1.4. The steps for generating mutant structures

In order to predict the binding affinity changes upon mutations, we minimize the wild-type structures, and generate the mutant models using FoldX v5.0 (Delgado *et al.*, 2019) according to the following steps (Mitra *et al.*, 2013):

(1) For $\Delta\Delta G_{\text{wt}\to\text{mt}}$ predictions, we fetch PDB file of the crystal structure (e.g., `wildtype.pdb`) from Protein Data Bank (PDB) (wwPDB consortium, 2019), and use `RepairPDB` command of FoldX to identify and repair those residues which have bad torsion angles, Vander Waals' clashes, or total energy as follows:

```
./FoldX --command=RepairPDB
--pdb=wildtype.pdb
```

After this step, we achieve the output file named `wildtype_Repair.pdb` and this structure is then used as the initial model to build the mutant model.

(2) We build the mutant model by using `BuildModel` command of FoldX as follows:

```
./FoldX --command=BuildModel
--pdb=wildtype_Repair.pdb
--mutant-file=individual-list.txt
```

Algorithm 1: Algorithm for CATH homologous superfamily based dataset splitting

Input : SKEMPI v2.0 dataset $\mathcal{D}_{\text{skempi2}}$, CATH-domain-list $\texttt{Domain}_{\text{cath}}$.
Output: SKEMPI v2.0 dataset splitting: $\{S_{\text{train}}, S_{\text{test}}\}$.

Construct chain set $\mathcal{C}_{\text{skempi2}}$ for SKEMPI v2.0 $\mathcal{C}_{\text{skempi2}} \leftarrow \text{skempi2\_chainset}(\mathcal{D}_{\text{skempi2}})$;
Initialize node set $\mathcal{V} \leftarrow \emptyset$, edge set $E \leftarrow \emptyset$;
foreach *chain* $c \in \mathcal{C}_{skempi2}$ **do**
  $d \leftarrow \text{cath\_domain\_stat}(c, \texttt{Domain}_{\text{cath}})$;
  if $d \neq 0.0.0.0$ **then**
    $\mathcal{V} \leftarrow \mathcal{V} \cup \{d\}$;
  else
    Record c in zero-domain-list $\texttt{Domain}_{\text{zero}}$;
  end
end
foreach *chain* $c \in \texttt{Domain}_{zero}$ **do**
  if $c \in \texttt{Foldseek}$ **then**
    $\texttt{Domain}_{\text{zero}} \leftarrow \texttt{Domain}_{\text{zero}} \setminus \{c\}$;
    $c \leftarrow \textbf{UPDATE}(\texttt{Foldseek}, c)$;
    $\texttt{Domain}_{\text{cath}} \leftarrow \texttt{Domain}_{\text{cath}} \cup \{c\}$;
  end
end
foreach *complex* $p \in \mathcal{D}_{skempi2}$ **do**
  $\mathcal{D}_p \leftarrow \text{ComplexDomains}\,(p, \mathcal{C}_{\text{skempi2}}, \texttt{Domain}_{\text{cath}}, \texttt{Domain}_{\text{zero}})$;
  foreach $d_i, d_j \in \mathcal{D}_p \times \mathcal{D}_p$ *where* $i < j$ **do**
    $\mathcal{E} \leftarrow \mathcal{E} \cup \{(d_i, d_j)\}$;
  end
end
Build CATH homologous superfamily graph $\mathcal{G} \leftarrow (\mathcal{V}, \mathcal{E})$;
$\mathcal{C}_{\mathcal{G}} \leftarrow \text{connected\_components}(\mathcal{G})$;
Sort $\mathcal{C}_{\mathcal{G}}$ by $|c_{\mathcal{G}}|$ in descending order ;
$\text{SIZE}_{\text{train}} \leftarrow \lfloor 0.7 \times |\mathcal{V}| \rfloor$;
Initialize $S_{\text{train}} \leftarrow \emptyset$, $S_{\text{test}} \leftarrow \emptyset$;
foreach *component* $c_{\mathcal{G}} \in \mathcal{C}_{\mathcal{G}}$ **do**
  if $|S_{train}| + |c_{\mathcal{G}}| \leq \text{SIZE}_{train}$ **then**
    $S_{\text{train}} \leftarrow S_{\text{train}} \cup c_{\mathcal{G}}$;
  else
    $S_{\text{test}} \leftarrow S_{\text{test}} \cup c_{\mathcal{G}}$;
  end
end
return $S_{\text{train}}, S_{\text{test}}$

Here, `individual_list.txt` contains the specified format of mutation(s), which can be either single or multiple mutations. After this step, two files are generated, including `WT_wildtype_Repair_1.pdb` and

Table S1. Performance comparison between different hyper-parameter settings for β under all mutations on held-out CATH test set, where bold value indicates the best performance under each metric.

Hyper-parameter	Overall					Per-PPI	
	PearsonR↑	SpearmanR↑	RMSE↓	MAE↓	AUROC↑	PearsonR↑	SpearmanR↑
$\beta = 0.6$	**0.6150**	**0.6269**	**2.0504**	**1.5047**	0.7805	**0.5260**	**0.4940**
$\beta = 0.4$	0.5934	0.5992	2.0930	1.5632	0.7848	0.5029	0.4480
$\beta = 0.8$	0.6145	0.6075	2.0514	1.5265	**0.7869**	0.5197	0.4631

Table S2. Performance comparison of three-fold cross-validation under single, multiple and all mutations on SKEMPI2 v2.0 dataset, where bold value indicates the best performance under each metric. In this experiment, we use the same split setting of training and test set as in RDE-Network.

Method	Mutations	Overall					per-PPI	
		PearsonR↑	SpearmanR↑	RMSE↓	MAE↓	AUROC↑	PearsonR↑	SpearmanR↑
DDGPred[a]	all	0.6580	0.4687	1.4998	1.0821	0.6992	0.3750	0.3407
	single	0.6515	0.4390	1.3285	0.9618	0.6858	0.3711	0.3427
	multiple	0.5938	0.5150	2.1813	1.6699	0.7590	0.3912	0.3896
End-to-End[a]	all	0.6373	0.4882	1.6198	1.1761	0.7172	0.3873	0.3587
	single	0.6605	0.4594	1.3148	0.9569	0.7019	0.3818	0.3426
	multiple	0.5858	0.4942	2.1971	1.7087	0.7532	0.4178	0.4034
MIF-Network[a]	all	0.6523	0.5134	1.5932	1.1469	0.7329	0.3965	0.3509
	single	0.6667	0.4802	1.3052	0.9411	0.7175	0.3952	0.3479
	multiple	0.6139	0.5370	2.1399	1.6422	0.7735	0.3968	0.3789
RDE-Network[a]	all	0.6447	0.5584	1.5799	1.1123	0.7454	0.4448	0.4010
	single	0.6421	0.5271	1.3333	0.9392	0.7367	0.4687	0.4333
	multiple	0.6288	0.5900	2.0980	1.5747	0.7749	0.4233	0.3926
DiffAffinity[a]	all	0.6690	0.5560	1.5350	1.0930	0.7440	0.4220	0.3970
	single	0.6720	0.5230	1.2880	0.9230	0.7330	0.4290	0.4090
	multiple	0.6500	0.6020	2.0510	1.5400	0.7840	0.4140	0.3870
Prompt-DDG[a]	all	0.6772	0.5910	1.5207	1.0770	0.7568	0.4712	0.4257
	single	0.6596	0.5450	1.3072	0.9191	0.7355	0.4736	0.4392
	multiple	0.6780	0.6433	1.9831	1.4837	0.8187	0.4448	0.3961
CATH-ddG	all	**0.7151**	**0.6393**	**1.4447**	**1.0335**	**0.7760**	**0.5251**	**0.4600**
	single	**0.6840**	**0.5862**	**1.2688**	**0.8998**	**0.7532**	**0.5236**	**0.4588**
	multiple	**0.7308**	**0.7142**	**1.8419**	**1.3958**	**0.8402**	**0.5079**	**0.4807**

[a] Results are from Prompt-DDG (Wu *et al.*, 2024).

`wildtype_Repair_1.pdb`. The first represents the wild-type structure with additional optimization, while the second corresponds to the mutant structure. Commonly, `WT_wildtype_Repair_1.pdb` is identical to `wildtype_Repair_1.pdb`, and if not, their differences are usually negligible.

(3) We compute the binding affinity of wild-type and mutant structure, respectively, by utilizing `AnalyseComplex` command of FoldX as follows:

```
./FoldX --command=AnalyseComplex
--pdb=WT_wildtype_Repair_1.pdb
--analyseComplexChains=Mol1,Mol2

./FoldX --command=AnalyseComplex
--pdb=wildtype_Repair_1.pdb
--analyseComplexChains=Mol1,Mol2
```

where `Mol1`, `Mol2` means the chains `Mol1` against `Mol2` that determine the interaction energy of the

Table S3. Performance comparison between different hyper-parameter settings for γ under all mutations on held-out CATH test set, where bold value indicates the best performance under each metric.

Hyper-parameter	Overall					Per-PPI	
	PearsonR↑	SpearmanR↑	RMSE↓	MAE↓	AUROC↑	PearsonR↑	SpearmanR↑
$\gamma = 0.5$	**0.6150**	**0.6269**	**2.0504**	**1.5047**	0.7805	**0.5260**	**0.4940**
$\gamma = 0.0$	0.5818	0.5998	2.1147	1.5778	0.7709	0.5091	0.4391
$\gamma = 0.2$	0.5929	0.5966	2.0940	1.5595	0.7817	0.5094	0.4570
$\gamma = 0.4$	0.5991	0.6152	2.0819	1.5416	0.7917	0.5101	0.4438
$\gamma = 0.6$	0.6034	0.6113	2.0735	1.5404	0.7889	0.5232	0.4643
$\gamma = 0.8$	0.6037	0.6095	2.0729	1.5485	**0.8026**	0.4810	0.4208
$\gamma = 1.0$	0.6048	0.6068	2.0707	1.5576	0.7888	0.5060	0.4296

protein complex. After this step, we calculate the change in binding affinity between wild-type and mutant from `Interaction Energy` term in `Interaction_wildtype_Repair_1_AC.fxout` and `Interaction_WT_wildtype_Repair_1_AC.fxout`. The energy terms in the output file that reflect the respective energy changes upon binding, including `Backbone Hbond`, `side-chain Hbond`, etc., are used as inputs to $\text{MLP}(\cdot)$ predictor of the FoldX module (see Figure2(a)).

(4) Finally, we leverage the `Optimize` command of FoldX to optimize the mutant structure for eliminating the Van der Waals' clashes by slightly moving all side-chains as follows:

```
./FoldX --command=Optimize
--pdb=wildtype_Repair_1.pdb
```

After this step, the output file `Optimized_wildtype_Repair_1.pdb` as well as `WT_wildtype_Repair_1.pdb` generated in the second step were used as the mutant and wild-type structural inputs of our model, respectively.

1.5. Visualization of superfamily distribution between CATH v4.3 and SKEMPI v2.0

In addition, to more comprehensively demonstrate the distinct differences in protein domain distribution between the CATH v4.3 database and the SKEMPI v2.0 dataset, we have visualized the detailed annotation distribution of class, architecture, topology, and homologous superfamily in Figure S2. This visualization provides a clear and intuitive comparison that highlights the characteristics in the domain organization of proteins.

1.6. Hyper-parameter selection of γ

In Equation 22, we set the weight hyper-parameter of the CATH domain loss function to $\gamma = 0.5$ for our initial experiments. In this section, we explore the effects of varying the weight γ of CATH loss by conducting comparative experiments with different values. The results are shown in Table S3.

1.7. Detailed prediction results under different weight settings

TThe following Table S4 provides a comprehensive overview of the differences in prediction performance of CATH-ddG under various weight settings between structure-based and energy-based predictive results during the inference process.

1.8. Visualization of rigid and flexible components of molecular

In addition, to demonstrate the rigid backbone and flexible side-chain components of the protein molecular, we visualize the fragment structure of the protein with PDB ID: 3EQS, as shown in Figure S3.

1.9. Hyper-parameter selection of p

In Section 3.6, we rotate the side-chain dihedral angles $180°$ uniformly at random with probability $p = 0.2$ in our experiments. In this section, we provide comparative experiments by setting the probability for flipping p to different values, see Table S10.

1.10. Details of baseline methods

Based on the input requirements of various baseline approaches, these methods can be classified into three categories, including structure-only methods (FoldX, flex ddG, ESM-IF, and ProteinMPNN), sequence and structure-based methods (RDE-Network, DiffAffinity, Prompt-DDG,

Table S4. Comparison of predictive performance with different weight settings between structure-based task and energy-based task during inference.

Structure-based	Energy-based	Mutations	Overall					per-PPI	
			PearsonR↑	SpearmanR↑	RMSE↓	MAE↓	AUROC↑	PearsonR↑	SpearmanR↑
0.0	1.0	all	0.5196	0.5774	2.2216	1.6235	0.7647	0.5204	0.4548
		single	0.5327	0.5521	1.6546	1.2310	0.7275	0.5527	0.4966
		multiple	0.4564	0.3972	3.0584	2.4144	0.8147	0.6204	0.5718
0.1	0.9	all	0.5418	0.5924	2.1855	1.5979	0.7702	0.5302	0.4546
		single	0.5565	0.5651	1.6245	1.2090	0.7336	0.5661	0.4963
		multiple	0.4825	0.4269	3.0108	2.3824	0.8209	0.6226	0.5716
0.2	0.8	all	0.5635	0.6057	2.1481	1.5723	0.7755	0.5395	0.4866
		single	0.5798	0.5764	1.5930	1.1863	0.7404	0.5788	0.5072
		multiple	0.5080	0.4570	2.9607	2.3534	0.8256	**0.6230**	**0.5748**
0.3	0.7	all	0.5847	0.6178	2.1095	1.546	0.7806	**0.5411**	0.4870
		single	0.6011	0.5892	1.5625	1.1631	0.7476	**0.5877**	**0.5114**
		multiple	0.5339	0.4849	2.9064	2.3192	0.8293	0.6186	0.5501
0.4	0.6	all	0.6005	0.6252	2.0792	1.5230	**0.7811**	0.5400	0.4868
		single	0.6158	**0.5943**	1.5405	1.1456	0.7473	0.5815	0.5022
		multiple	0.5539	0.5087	2.8618	2.2886	0.8303	0.6089	0.5386
0.5	0.5	all	0.6150	**0.6269**	2.0504	1.5047	0.7805	0.5260	**0.4940**
		single	**0.6250**	0.5937	**1.5263**	**1.1377**	0.7464	0.5690	0.5090
		multiple	0.5746	0.5275	2.8131	2.2518	0.8325	0.5965	0.5507
0.6	0.4	all	0.6212	0.6223	2.0377	1.4994	0.7799	0.5080	0.4841
		single	0.6236	0.5843	1.5285	1.1417	0.7462	0.5560	0.4868
		multiple	0.5866	0.5432	2.7838	2.2342	0.8288	0.5732	0.5369
0.7	0.3	all	**0.6262**	0.6167	**2.0272**	**1.4920**	0.7804	0.4815	0.4432
		single	0.6202	0.5771	1.5337	1.1505	**0.7483**	0.5255	0.4541
		multiple	0.5969	0.5537	2.7578	2.1957	**0.8327**	0.5468	0.5167
0.8	0.2	all	0.6198	0.5934	2.0406	1.5077	0.7704	0.4454	0.4227
		single	0.5932	0.5451	1.5740	1.1801	0.7408	0.4864	0.4445
		multiple	**0.6022**	0.5568	**2.7442**	2.1851	0.8245	0.5255	0.4960
0.9	0.1	all	0.6122	0.5732	2.0559	1.5256	0.7641	0.4168	0.3906
		single	0.5749	0.5141	1.5997	1.2079	0.7334	0.4565	0.4050
		multiple	0.6004	**0.5575**	2.7488	2.1790	0.8207	0.4844	0.4668
1.0	0.0	all	0.5963	0.5399	2.0873	1.5551	0.7497	0.3678	0.3608
		single	0.5369	0.4732	1.6495	1.2487	0.7234	0.4012	0.3734
		multiple	0.5987	0.5527	2.7533	**2.1765**	0.8074	0.4650	0.4787

PPIFORMER, GearBind, and DDAffinity), and sequence alignment-based method (GEMME).

(1) FoldX (Delgado *et al.*, 2019) is a widely utilized computational tool that employs a force-field-based approach

to predict changes in protein stability or binding affinity ($\Delta\Delta G$) resulting from mutations. The algorithm incorporates different physical energy terms, including van der Waals interactions, hydrogen bonding, electrostatic interactions, and solvation effects, which have been weighted with empirical data obtained from experimental protein structures. In its computational process, FoldX optimizes the mutant protein structure based on the wild-type and subsequently performs a precise calculation of the energy difference between the mutant and wild-type structures.

(2) The flex ddG module (Barlow *et al.*, 2018), integrated within the Rosetta macromolecular modeling suite, is specifically designed for predicting binding affinity changes through explicit modeling of protein conformational flexibility. Unlike FoldX, which focuses on rapid energy-based calculations, flex ddG employs detailed molecular dynamics and Monte Carlo sampling of conformational changes, particularly in the mutated region. While FoldX prioritizes computational efficiency for high throughput mutagenesis studies, flex ddG achieves higher prediction accuracy through rigorous conformational sampling, albeit at the cost of requiring more computational resources.

(3) ESM-IF (Hsu *et al.*, 2022) and ProteinMPNN (Dauparas *et al.*, 2022) both tackle the inverse folding problem by using the backbone atomic coordinates of protein 3D structures as input to guide sequence design, aiming to generate amino acid sequences that match a given structure. ESM-IF is a conditional language model that models the conditional probability distribution of a sequence, using the protein structure as condition. Specifically, given the protein backbone structure, the ESM-IF predicts the likelihood probability of the amino acid sequence as the mutation effect prediction score. In contrast, ProteinMPNN employs a Graph Neural Network (GNN) architecture, utilizing message passing between nodes and edges to model the interactions within the protein structures.

(4) Sequence and structure-based pre-training based methods fine-tuned on $\Delta\Delta G$ labels, including RDE-Network (Luo *et al.*, 2023), DiffAffinity (Liu *et al.*, 2023), Prompt-DDG (Wu *et al.*, 2024), PPIFORMER (Bushuiev *et al.*, 2024), and GearBind (Cai *et al.*, 2024). End-to-end learning models, such as DDAffinity (Yu *et al.*, 2024), employ innovative architectures to directly predict $\Delta\Delta G$ values. Specifically, DDAffinity leverages the sequential and spatial encoder from ProteinMPNN as the backbone for encoding, and further incorporates a weighted sharing multilayer perceptron (MLP) to achieve $\Delta\Delta G$ prediction. This integrated approach enables the model to capture relationships between protein sequences and structures, thereby enhancing the accuracy and efficiency of $\Delta\Delta G$ prediction.

(5) Global Epistatic Model for predicting Mutational Effects (GEMME), developed by Laine *et al.* (2019), represents a sequence-based prediction approach that leverages evolutionary information from multiple sequence alignments (MSAs) to estimate the effects of mutations. This innovative method specifically models the evolutionary information of protein sequences and calculates variant scores through the analysis of amino acid conservation patterns. Notably, GEMME has demonstrated exceptional performance, currently ranking as the tenth in the ProteinGym benchmark (Notin *et al.*, 2023). This benchmark is specifically designed to evaluate model performance in predicting protein fitness from deep mutational scanning (DMS) data.

Table S5. Performance comparison of CATH-ddG on held-out protein test set (Complement C3d and Fibrinogen-binding protein Efb-C) from SKEMPI v2.0 dataset.

Method	SpearmanR↑	Precision↑	Recall↑
FoldX[b]	0.68	**100.00%**	**100.00%**
flex ddG[a]	0.68	**100.00%**	50.00%
GEMME[a]	0.61	**100.00%**	**100.00%**
MSA Transformer[a]	0.61	**100.00%**	**100.00%**
ESM-IF[a]	0.34	50.00%	50.00%
RDE-Network[a]	0.68	**100.00%**	**100.00%**
PPIFORMER[a]	**0.75**	**100.00%**	**100.00%**
Prompt-DDG[b]	0.61	0.00%	0.00%
DiffAffinity[b]	0.61	**100.00%**	50.00%
DDAffinity[b]	0.58	76.92%	83.33%
CATH-ddG	0.63	**100.00%**	**100.00%**

[a] Results are from PPIFORMER Bushuiev *et al.* (2024).
[b] Results are from released tool or source code.

1.11. Computational efficiency analysis of CATH-ddG

In terms of computational requirements for training, we provide a comprehensive analysis of the computational complexity metrics for CATH-ddG, including both the scale of trainable parameters (#Params) and the number of floating point operations (FLOPs) involved in the training process. Specifically, CATH-ddG consists of approximately 6.59 M trainable parameters and requires around 19.7 G FLOPs per forward pass. The training process, which is conducted on the SKEMPI v2.0 dataset with the held-out CATH dataset as the test set, is completed in approximately 3.03 hours. During this training phase, the NVIDIA A100 GPU utilizes around 19.7 GB of memory.

For the computational requirements during inference, we first count the sequence length distribution of protein com-

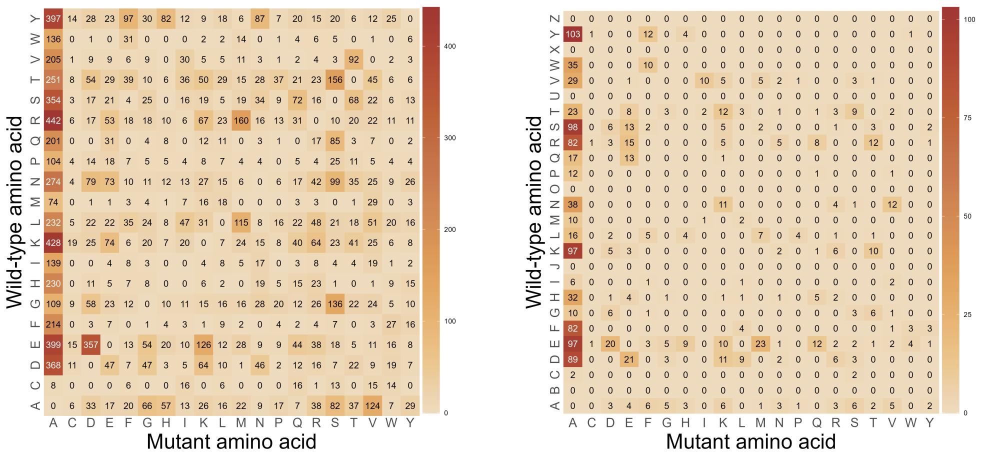

(a) Mutation distribution of SKEMPI v2.0 **(b)** Mutation distribution of held-out CATH test set

Fig. S1. Comparison of mutation distribution between the SKEMPI v2.0 dataset and the held-out CATH test set. Alanine mutations constitute a substantial proportion of the total mutations in both datasets, with a notably higher prevalence observed in the held-out CATH test set. This comparison highlights the potential bias toward alanine mutations, which may influence the evaluation of predictive performance.

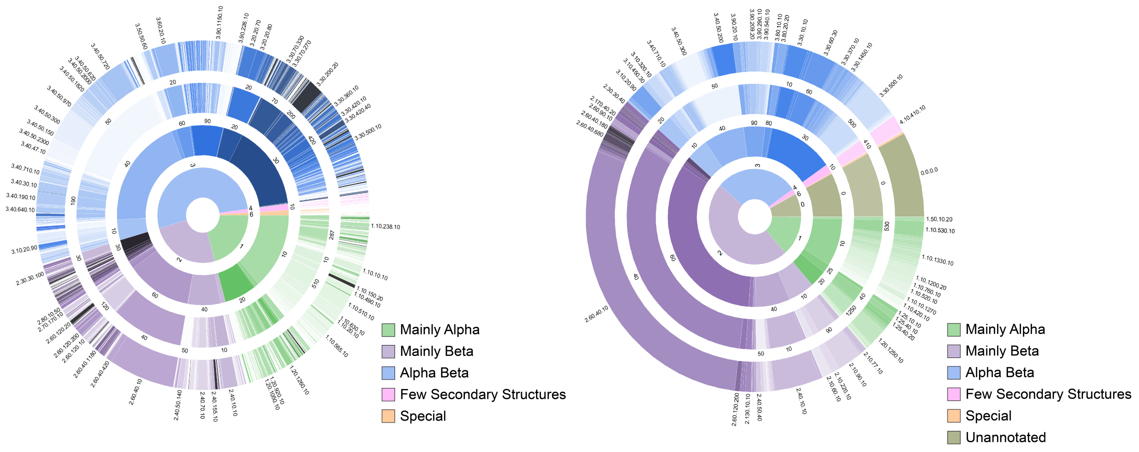

(a) CATH v4.3 (b) SKEMPI v2.0

Fig. S2. An illustration of protein domain distribution for CATH v4.3 and SKEMPI v2.0.

plexes in the SKEMPI2 dataset, as shown in Figure S4. Among them, the shortest sequence length is 57, the longest sequence length is 3397, and the proportion of sequence lengths less than 1000 is 95%. Second, we analyze the time efficiency analysis involved in model inference, including the following steps: RepairPDB, BuildModel, Optimize, AnalyseComplex, Structure generation, ESM2 embeddings and Inference. As shown in the table, the RepairPDB step involves repairing bad torsional angles, Van der Waals' clashes, etc. Considering that the RepairPDB step needs to be performed only once for each protein complex structure, it is negligible for the total time efficiency assessment of the screening process. Finally, we evaluate the time efficiency analysis of single and multiple mutations for the shortest, longest, and intermediate sequence length, respectively, as shown in Table S11.

We also evaluate the time efficiency analysis of single point mutations and multiple mutations for the shortest, longest, and intermediate sequence length, respectively, and the computational efficiency analysis of flex ddG are listed in Table S12. For each mutation, $\Delta\Delta G$ predictions are obtained by averaging the 5 results of $\Delta\Delta G$ predictions for each mutation, and the default parameter settings for flex ddG are as follows:

```
'-s %s' % input_pdb_path,
'-parser:protocol', 'ddG-backrub.xml',
'-parser:script_vars',
'chainstomove=' + chains_to_move,
```

Table S6. Performance comparison of CATH-ddG on held-out protein test set (Barnase and barstar) from SKEMPI v2.0 dataset.

Method	SpearmanR↑	Precision↑	Recall↑
FoldX[b]	0.72	34.38%	**78.57%**
flex ddG[a]	0.82	42.86%	42.86%
GEMME[a]	0.40	**100.00%**	64.29%
MSA Transformer[a]	0.43	87.50%	50.00%
ESM-IF[a]	0.18	41.18%	50.00%
RDE-Network[a]	0.58	42.11%	57.14%
PPIFORMER[a]	0.60	38.46%	71.43%
Prompt-DDG[b]	0.56	37.50%	42.86%
DiffAffinity[b]	0.63	39.29%	**78.57%**
DDAffinity[b]	0.63	43.94%	69.05%
CATH-ddG	**0.84**	55.56%	71.43%

[a] Results are from PPIFORMER Bushuiev *et al.* (2024)
[b] Results are from released tool or source code.

Table S7. Performance comparison of CATH-ddG on held-out protein test set (C. thermophilum YTM1 and C. thermophilum ERB1) from SKEMPI v2.0 dataset.

Method	SpearmanR↑	Precision↑	Recall↑
FoldX[b]	0.93	83.33%	**100.00%**
flex ddG[a]	**0.98**	**100.00%**	**100.00%**
GEMME[a]	0.79	**100.00%**	80.00%
MSA Transformer[a]	0.05	66.67%	40.00%
ESM-IF[a]	0.09	0.00%	0.00%
RDE-Network[a]	0.15	50.00%	40.00%
PPIFORMER[a]	0.34	60.00%	60.00%
Prompt-DDG[b]	-0.25	0.00%	0.00%
DiffAffinity[b]	0.11	50.00%	40.00%
DDAffinity[b]	0.27	64.29%	60.00%
CATH-ddG	0.77	83.33%	**100.00%**

[a] Results are from PPIFORMER Bushuiev *et al.* (2024)
[b] Results are from released tool or source code.

```
'mutate_resfile_relpath=' +
'nataa_mutations.resfile',
'number_backrub_trials=%d' % 35000,
'max_minimization_iter=%d' % 5000,
'abs_score_convergence_thresh=%.1f' % 1.0,
'backrub_trajectory_stride=%d' % 7000,
'-restore_talaris_behavior',
'-in:file:fullatom',
'-ignore_unrecognized_res',
'-ignore_zero_occupancy false',
'-ex1',
'-ex2',
```

Table S8. Performance comparison of CATH-ddG on held-out protein test set (dHP1 Chromodomain and H3 tail) from SKEMPI v2.0 dataset.

Method	SpearmanR↑	Precision↑	Recall↑
FoldX[b]	-0.14	27.91%	85.71%
flex ddG[a]	-0.05	29.27%	85.71%
GEMME[a]	-0.10	0.00%	0.00%
MSA Transformer[a]	0.09	0.00%	0.00%
ESM-IF[a]	**0.10**	45.45%	71.43%
RDE-Network[a]	-0.40	30.23%	**92.86%**
PPIFORMER[a]	0.00	**53.33%**	57.14%
Prompt-DDG[b]	-0.27	17.86%	35.71%
DiffAffinity[b]	-0.20	30.30%	71.43%
DDAffinity[b]	-0.07	31.58%	57.14%
CATH-ddG	-0.07	25.00%	57.14%

[a] Results are from PPIFORMER Bushuiev *et al.* (2024)
[b] Results are from released tool or source code.

Table S9. Performance comparison of CATH-ddG on held-out protein test set (E6AP and UBCH7) from SKEMPI v2.0 dataset.

Method	SpearmanR↑	Precision↑	Recall↑
FoldX[b]	0.48	41.67%	**41.67%**
flex ddG[a]	0.29	44.44%	33.33%
GEMME[a]	0.20	0.00%	0.00%
MSA Transformer[a]	0.37	0.00%	0.00%
ESM-IF[a]	0.21	30.77%	33.33%
RDE-Network[a]	0.21	50.00%	33.33%
PPIFORMER[a]	0.43	40.00%	16.67%
Prompt-DDG[b]	-0.07	0.00%	0.00%
DiffAffinity[b]	0.42	60.00%	25.00%
DDAffinity[b]	0.30	40.00%	16.67%
CATH-ddG	**0.61**	**80.00%**	33.33%

[a] Results are from PPIFORMER Bushuiev *et al.* (2024).
[b] Results are from released tool or source code.

1.12. Detailed results under PPIFORMER data splitting

Table 3 shows the average performance of all compared methods on 5 held-out proteins from SKEMPI v2.0. In this section, we provide a more detailed comparison. Specifically, Table S5-S9 demonstrate non-aggregated performance of baseline methods and CATH-ddG on all held-out proteins. We use ChimeraX v1.8 (Goddard *et al.*, 2018) to visualize protein complexes from protein groups, where the mutation sites are colored in blue, as in Figure S5. We find that the predictive performance of the model decreases significantly when the mutation sites are located in disor-

Table S10. Performance comparison between different hyper-parameter settings for p under all mutations on held-out CATH test set, where bold value indicates the best performance under each metric.

Hyper-parameter	Overall					Per-PPI	
	PearsonR↑	SpearmanR↑	RMSE↓	MAE↓	AUROC↑	PearsonR↑	SpearmanR↑
$p = 0.2$	**0.6150**	**0.6269**	**2.0504**	**1.5047**	0.7805	**0.5260**	**0.4940**
$p = 0.1$	0.5815	0.5850	2.1154	1.5704	0.7765	0.4765	0.4421
$p = 0.3$	0.5957	0.5932	2.0885	1.5564	0.7795	0.4858	0.4450
$p = 0.4$	0.6099	0.6138	2.0607	1.5302	**0.7962**	0.5144	0.4733
$p = 0.5$	0.6007	0.6086	2.0788	1.5374	0.7890	0.5135	0.4511

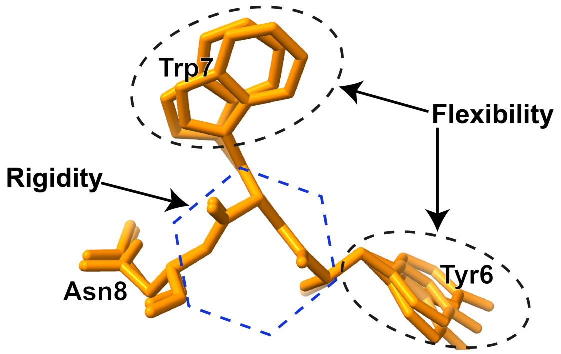

Fig. S3. Visualization of rigidity and flexibility in the fragment structure of the protein complex (PDB ID: 3EQS/B). Backbone bonds demonstrate near-rigidity, maintaining a stable and consistent conformation, while side-chain bonds exhibit greater flexibility, enabling dynamic and variable spatial configurations. This distinction highlights the structural stability of the protein backbone compared to the adaptable nature of side chains.

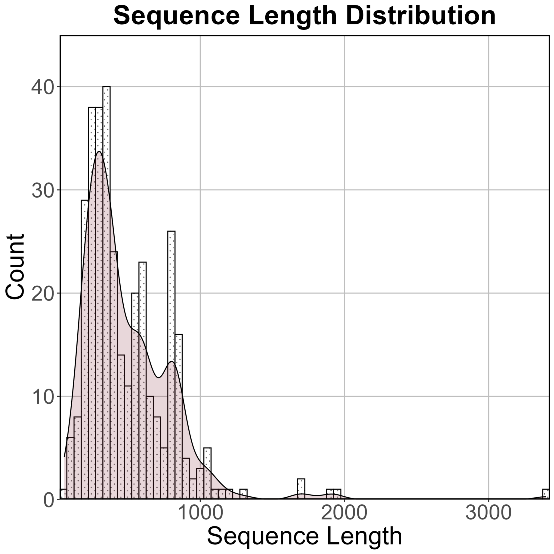

Fig. S4. Sequence length distribution of SKEMPI v2.0 dataset.

dered regions of the proteins that lack stable 3D structures, as illustrated in Figure S5 (d) and (e).

1.13. Detailed results under RDE-Network data splitting

We demonstrate the 3-fold cross-validation performance of CATH-ddG by utilizing the same data splitting of RDE-Network on SKEMPI v2.0 in Table S2. CATH-ddG outperforms all baseline methods in all evaluation metrics. In particular, CATH-ddG achieves the improvement on PersonR of 5.60%, SpearmanR of 8.17%, minimized RMSE of 3.67%, miminzed MAE of 4.04%, AUROC of 2.54%, per-PPI PersonR of 11.44%, and per-PPI SpearmanR of 8.06% on all mutation entries, respectively, which demonstrates its significant advantages relative to baseline methods. Notably, when comparing the 3-fold cross-validation test set with the training set under the randomized data splitting on PPIs according to the RDE-Network, on average 88.70% of the mutation entries are classified as easy mutations, where a mutation is defined as easy its maximum TM-score ≥ 0.6 when compared to the training set.

1.14. Detailed results on HER2 test set

Table 4 shows the average performance of models trained with 3-fold cross-validation on SKEMPI v2.0 for all compared methods. Specifically, in this section, we provide a more detailed comparison in Table S13-S15, which demonstrate non-averaged performance of the methods on 3 test folds, separately.

1.15. Results of CATH-ddG with AlphaFold3 predicted structures

In addition, to illustrate the predictive performance of CATH-ddG with AlphaFold3 predicted structures as inputs, we conduct the experiments on held-out CATH test set using CATH-ddG trained on the experimentally derived PDB structures, and the results are shown in Table S16. The results indicate that our model demonstrates robust performance on the held-out CAHT test set when using Al-

Table S11. Computational efficiency analysis of CATH-ddG.

PDB ID	Length of Sequence	Number of mutations	Tool	Step	Time (ms)	Total time (s)	Device
1KNE	57	1	FoldX	RepairPDB	32,360	32.360	Intel Xeon Gold 6230R
			FoldX	BuildModel	10,338	13.102	Intel Xeon Gold 6230R
			FoldX	Optimize	900		
			FoldX	AnalyseComplex	1,517		
			CATH-ddG	Structure generation	207		
			ESM2	ESM2 embeddings	33		NVIDIA A100 GPU
			CATH-ddG	Inference	107		
1N8Z	1015	1	FoldX	RepairPDB	452,332	452.332	Intel Xeon Gold 6230R
			FoldX	BuildModel	8,455	27.723	Intel Xeon Gold 6230R
			FoldX	Optimize	5,214		
			FoldX	AnalyseComplex	10,034		
			CATH-ddG	Structure generation	3,884		
			ESM2	ESM2 embeddings	33		NVIDIA A100 GPU
			CATH-ddG	Inference	103		
3VR6	3397	1	FoldX	RepairPDB	2,692,503	2,692.503	Intel Xeon Gold 6230R
			FoldX	BuildModel	42,139	92.221	Intel Xeon Gold 6230R
			FoldX	Optimize	15,963		
			FoldX	AnalyseComplex	23,860		
			CATH-ddG	Structure generation	10,078		
			ESM2	ESM2 embeddings	74		NVIDIA A100 GPU
			CATH-ddG	Inference	107		
1KNE	57	5	FoldX	RepairPDB	34,988	34.988	Intel Xeon Gold 6230R
			FoldX	BuildModel	72,156	75.571	Intel Xeon Gold 6230R
			FoldX	Optimize	1,135		
			FoldX	AnalyseComplex	1,920		
			CATH-ddG	Structure generation	217		
			ESM2	ESM2 embeddings	38		NVIDIA A100 GPU
			CATH-ddG	Inference	105		
1N8Z	1015	5	FoldX	RepairPDB	478,806	478.806	Intel Xeon Gold 6230R
			FoldX	BuildModel	101,771	122.742	Intel Xeon Gold 6230R
			FoldX	Optimize	6,156		
			FoldX	AnalyseComplex	10,905		
			CATH-ddG	Structure generation	3,773		
			ESM2	ESM2 embeddings	36		NVIDIA A100 GPU
			CATH-ddG	Inference	101		
3VR6	3397	5	FoldX	RepairPDB	2,805,940	2,805.940	Intel Xeon Gold 6230R
			FoldX	BuildModel	178,594	232.780	Intel Xeon Gold 6230R
			FoldX	Optimize	17,765		
			FoldX	AnalyseComplex	23,881		
			CATH-ddG	Structure generation	12,363		
			ESM2	ESM2 embeddings	72		NVIDIA A100 GPU
			CATH-ddG	Inference	105		

phaFold3 predicted structures as input, further validating its robustness and generalizability.

This finding suggests that our model is not only effective when trained and tested on high-resolution and experimen-

Table S12. Computational efficiency analysis of flex ddG.

PDB ID	Length of Sequence	Number of mutations	Total time (s)	Device
1KNE	57	1	671.82	Intel Xeon Gold 6230R
1N8Z	1015	1	68,723.36	
3VR6	3397	1	288,363.88	
1KNE	57	5	687.79	
1N8Z	1015	5	73,004.53	
3VR6	3397	5	301,529.41	

Table S13. The first fold prediction performance on HER2.

Number of mutations	Count	PearsonR↑	SpearmanR↑
2	18	0.574	0.544
10	41	0.052	0.080
8	87	0.603	0.550
9	70	0.482	0.488
7	75	0.561	0.577
5	24	0.786	0.566
11	24	0.217	0.237
12	11	0.720	0.636
4	10	0.738	0.600
6	52	0.662	0.538
3	4	0.831	0.200
13	2	1.000	1.000
1	1	–	–
Overall	419	0.575	0.603

Table S14. The second fold prediction performance on HER2.

Number of mutations	Count	PearsonR↑	SpearmanR↑
2	18	0.597	0.546
10	41	0.053	0.137
8	87	0.519	0.477
9	70	0.547	0.545
7	75	0.512	0.504
5	24	0.822	0.531
11	24	0.174	0.177
12	11	0.648	0.555
4	10	0.885	0.794
6	52	0.610	0.429
3	4	0.889	0.800
13	2	1.000	1.000
1	1	–	–
Overall	419	0.556	0.585

Table S15. The third fold prediction performance on HER2.

Number of mutations	Count	PearsonR↑	SpearmanR↑
2	18	0.653	0.610
10	41	0.301	0.304
8	87	0.596	0.594
9	70	0.561	0.560
7	75	0.582	0.579
5	24	0.749	0.529
11	24	0.311	0.317
12	11	0.823	0.936
4	10	0.896	0.818
6	52	0.638	0.508
3	4	0.840	0.800
13	2	1.000	1.000
1	1	–	–
Overall	419	0.608	0.640

tally derived PDB structures but also maintains its predictive accuracy when using computationally predicted structures. This capability is crucial for practical applications where high-resolution experimental data may not always be available. In future work, we will explore training prediction methods based on model-predicted structures to enhance the robustness and applicability of our CATH-ddG model. Specifically, we plan to incorporate AlphaFold-multimer (Evans *et al.*, 2021) predicted structures into our training datasets. These advancements will not only improve the performance in predicting the effects of mutations but also create new opportunities for practical applications in drug design and disease mechanism studies.

References

Barlow K. A, Ó Conchuír S, Thompson S *et al.* Flex ddG: Rosetta ensemble-based estimation of changes in protein–protein binding affinity upon mutation. *The Journal of Physical Chemistry B* 2018;**122:**5389–5399.

Bushuiev A, Bushuiev R, Kouba P *et al.* Learning to design protein-protein interactions with enhanced generalization. In *The Twelfth International Conference on Learning Representations, ICLR 2024, Vienna, Austria* 2024.

Cai H, Zhang Z, Wang M *et al.* Pretrainable geometric

Table S16. Blind test performance under single, multiple, and overall mutations on the held-out CATH test set using AlphaFold3 predicted structures as input.

Method	Mutations	Overall					per-PPI	
		PearsonR↑	SpearmanR↑	RMSE↓	MAE↓	AUROC↑	PearsonR↑	SpearmanR↑
CATH-ddG[a]	all	**0.6227**	0.6210	**2.0346**	**1.4950**	**0.7845**	0.4922	0.4235
	single	**0.6271**	0.5880	**1.5229**	1.1563	**0.7613**	**0.5818**	0.4940
	multiple	**0.5897**	**0.5479**	**2.7760**	**2.1872**	0.8259	0.5895	**0.5629**
CATH-ddG	all	0.6150	**0.6269**	2.0504	1.5047	0.7805	**0.5260**	**0.4940**
	single	0.6250	**0.5937**	1.5263	**1.1377**	0.7464	0.5690	**0.5090**
	multiple	0.5746	0.5275	2.8131	2.2518	**0.8325**	**0.5965**	0.5507

[a] Results are from using AlphaFold3 predicted structures as input.

graph neural network for antibody affinity maturation. *Nature communications* 2024;**15:**7785.

Dauparas J, Anishchenko I, Bennett N *et al.* Robust deep learning based protein sequence design using ProteinMPNN. *Science* 2022;**378:**49–56.

Delgado J, Radusky L. G, Cianferoni D *et al.* FoldX 5.0: working with RNA, small molecules and a new graphical interface. *Bioinformatics* 2019;**35:**4168–4169.

Evans R, O'Neill M, Pritzel A *et al.* Protein complex prediction with AlphaFold-Multimer. *biorxiv* 2021:2021–10.

Goddard T. D, Huang C. C, Meng E. C *et al.* UCSF ChimeraX: Meeting modern challenges in visualization and analysis. *Protein science* 2018;**27:**14–25.

Hsu C, Verkuil R, Liu J *et al.* Learning inverse folding from millions of predicted structures. In *International Conference on Machine Learning, ICML 2022, Baltimore, Maryland, USA* 2022; 8946–8970.

Laine E, Karami Y and Carbone A. GEMME: a simple and fast global epistatic model predicting mutational effects. *Molecular biology and evolution* 2019;**36:**2604–2619.

Liu S, Zhu T, Ren M *et al.* Predicting mutational effects on protein-protein binding via a side-chain diffusion probabilistic model. In *Advances in Neural Information Processing Systems 36: Annual Conference on Neural Information Processing Systems, NeurIPS 2023, New Orleans, LA, USA* 2023.

Luo S, Su Y, Wu Z *et al.* Rotamer density estimator is an unsupervised learner of the effect of mutations on protein-protein interaction. In *The Eleventh International Conference on Learning Representations, ICLR 2023, Kigali, Rwanda* 2023.

Mitra P, Shultis D and Zhang Y. EvoDesign: de novo protein design based on structural and evolutionary profiles. *Nucleic acids research* 2013;**41:**W273–W280.

Notin P, Kollasch A, Ritter D *et al.* Proteingym: Large-scale benchmarks for protein fitness prediction and design. *Advances in Neural Information Processing Systems* 2023;**36:**64331–64379.

Wu L, Tian Y, Lin H *et al.* Learning to predict mutational effects of protein-protein interactions by microenvironment-aware hierarchical prompt learning. In *Forty-first International Conference on Machine Learning, ICML 2024, Vienna, Austria* 2024.

wwPDB consortium. Protein Data Bank: the single global archive for 3D macromolecular structure data. *Nucleic acids research* 2019;**47:**D520–D528.

Yu G, Zhao Q, Bi X *et al.* DDAffinity: predicting the changes in binding affinity of multiple point mutations using protein 3D structure. *Bioinformatics* 2024;**40:**i418–i427.

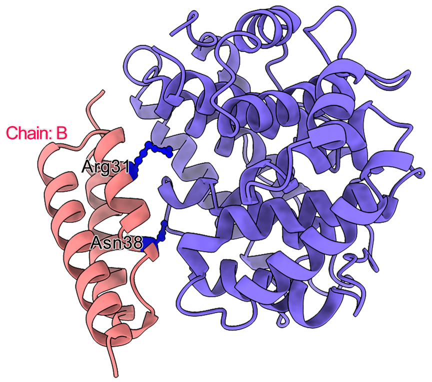

(a) Table S5: Complement C3d and Fibrinogen-binding protein Efb-C (PDB ID: 2GOX)

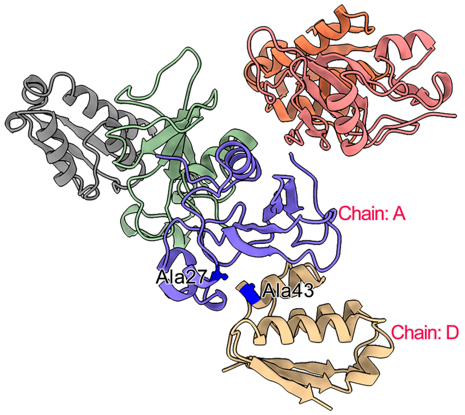

(b) Table S6: Barnase and barstar (PDB ID: 1B2S)

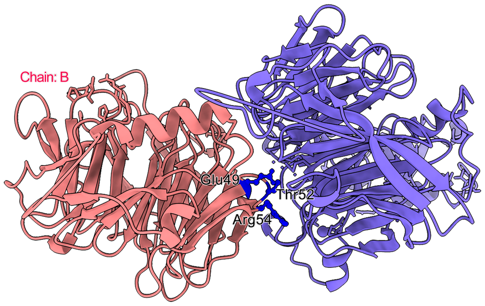

(c) Table S7: C. thermophilum YTM1 and C. thermophilum ERB1 (PDB ID: 5CXB)

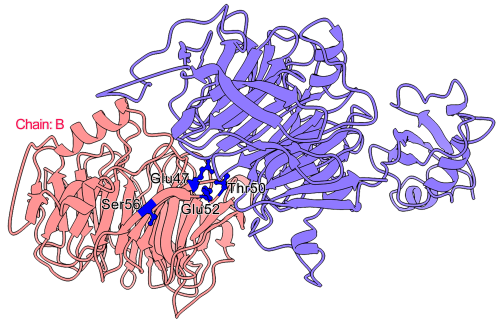

(d) Table S7: C. thermophilum YTM1 and C. thermophilum ERB1 (PDB ID: 5CYK)

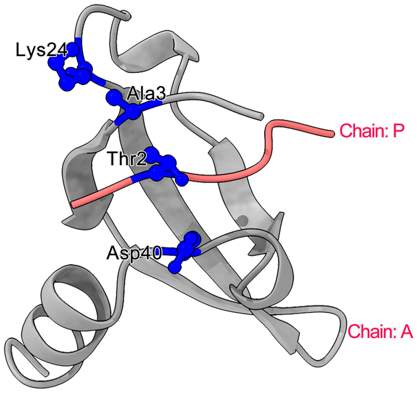

(e) Table S8: dHP1 Chromodomain and H3 tail (PDB ID: 1KNE)

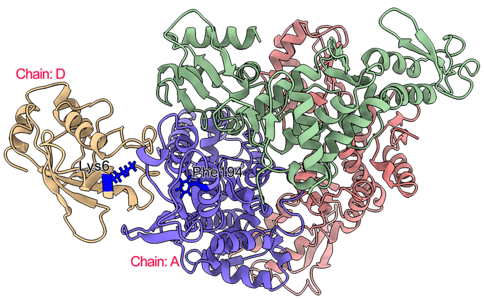

(f) Table S9: E6AP and UBCH7 (PDB ID: 1C4Z)

Fig. S5. Visualization of mutation sites for different protein groups. Mutation sites are highlighted in blue. In (e), all mutation sites of the displayed protein complex are located within disordered regions, potentially explaining the model's reduced predictive performance (see Table S8). A similar pattern is observed in (d), where some mutation sites also reside in disordered regions, further supporting the impact of structural disorder on prediction accuracy.
